# Synthesis, Properties, and Biomedical Application of Dicationic Gemini Surfactants with Dodecane Spacer and Carbamate Fragments

**DOI:** 10.3390/ijms241512312

**Published:** 2023-08-01

**Authors:** Leysan Vasileva, Gulnara Gaynanova, Farida Valeeva, Elvira Romanova, Rais Pavlov, Denis Kuznetsov, Grigory Belyaev, Irina Zueva, Anna Lyubina, Alexandra Voloshina, Konstantin Petrov, Lucia Zakharova

**Affiliations:** Arbuzov Institute of Organic and Physical Chemistry, FRC Kazan Scientific Center, Russian Academy of Sciences, 8 Arbuzov Str., 420088 Kazan, Russia

**Keywords:** gemini surfactant, aggregation, antimicrobial activity, liposome, α-tocopherol, donepezil hydrochloride, Alzheimer’s disease

## Abstract

A synthesis procedure and aggregation properties of a new homologous series of dicationic gemini surfactants with a dodecane spacer and two carbamate fragments (N,N′-dialkyl-N,N′-bis(2-(ethylcarbamoyloxy)ethyl)-N,N′-dimethyldodecan-1,6-diammonium dibromide, n-12-n(Et), where n = 10, 12, 14) were comprehensively described. The critical micelle concentrations of gemini surfactants were obtained using tensiometry, conductometry, spectrophotometry, and fluorimetry. The thermodynamic parameters of adsorption and micellization, i.e., maximum surface excess (Г_max_), the surface area per surfactant molecule (A_min_), degree of counterion binding (β), and Gibbs free energy of micellization (∆G_mic_), were calculated. Functional activity of the surfactants, including the solubilizing capacity toward Orange OT and indomethacin, incorporation into the lipid bilayer, minimum inhibitory concentration, and minimum bactericidal and fungicidal concentrations, was determined. Synthesized gemini surfactants were further used for the modification of liposomes dual-loaded with α-tocopherol and donepezil hydrochloride for intranasal treatment of Alzheimer’s disease. The obtained liposomes have high stability (more than 5 months), a significant positive charge (approximately + 40 mV), and a high degree of encapsulation efficiency toward rhodamine B, α-tocopherol, and donepezil hydrochloride. Korsmeyer-Peppas, Higuchi, and first-order kinetic models were used to process the in vitro release curves of donepezil hydrochloride. Intranasal administration of liposomes loaded with α-tocopherol and donepezil hydrochloride for 21 days prevented memory impairment and decreased the number of Aβ plaques by 37.6%, 40.5%, and 72.6% in the entorhinal cortex, DG, and CA1 areas of the hippocampus of the brain of transgenic mice with Alzheimer’s disease model (APP/PS1) compared with untreated animals.

## 1. Introduction

The biomimetic approach has been successfully applied for nanomaterial design [1,2,3]. At the same time, many biomolecules have the ability to self-assemble into regular structures. Within this context, surfactants are unique molecules for both forming nanocontainers and modifying the properties of other carriers [4,5]. The wide practical application of surfactants is due to their ability to adsorb at the interface, spontaneously form aggregates above the critical micelle concentration (cmc), and solubilize hydrophobic compounds [6,7,8]. Although micellar solutions of nonionic surfactants (Tween 20, Tween 80, Triton X-100, Pluronics, etc.) are widely used to increase the solubility of medicines [9,10], the special affinity of cationic surfactants for negatively charged surfaces is the reason for the synthesis of new cationic amphiphiles with an optimum balance of beneficial features and toxicity [6]. The key ways to achieve this goal are the inclusion of biodegradable fragments in surfactant molecules [11,12], the synthesis of surfactants based on natural raw materials [13,14], and the use of gemini surfactants (GS) [15,16]. GS are usually characterized by a high charge density and low cmc values compared with classical amphiphiles. These factors largely determine the advantage of GS for use as building blocks for non-viral gene delivery systems [17,18,19]. The introduction of biodegradable fragments into the GS molecule allows us to “kill two birds with one stone” when dealing with the problem of reducing cationic surfactant toxicity [20,21,22].

The modification of liposomes with cationic surfactants is being actively developed [23,24,25]. This is explained by the fact that, as the most successful type of carriers in clinical practice [26,27,28], liposomes make it possible to reveal the areas of surfactant biomedical application at low concentrations. The natural composition of liposomes determines their biocompatibility and low toxicity, whereas the inner structure of liposomes composed of a water pool surrounded by lipid bilayer provides the encapsulation of both hydrophilic and hydrophobic drugs [4,29,30]. Substrate entry into liposomes leads to an increase in their solubility and bioavailability, protection from biodegradation, and increased cellular uptake [31,32]. Modification of liposomes by cationic surfactants can be considered an alternative to the use of cationic lipids, which are expensive and toxic [21,33,34]. A number of factors can influence the key physicochemical parameters of modified liposomes: (1) surfactant head group structure [23,35,36]; (2) surfactant alkyl tail length [36,37]; (3) surfactant degree of oligomerization (monomeric, gemini, or trimeric) [38,39,40]; and (4) the lipid/surfactant ratio [41,42]. There are examples of liposome modification with alkyltriphenylphosphonium surfactants [37,43,44,45], amino acid-based surfactants [46,47], imidazolium surfactants [43,48,49,50], pyrrolidinium surfactants [51,52,53], metallosurfactants [54], and GS [21,55,56,57,58].

There is a successful illustration of cationic liposome use for intranasal delivery [45,59,60]. Cationic nanoparticles have the ability to interact with nasal epithelial cells that have a negative charge, thereby prolonging the retention time of the drug in the nasal cavity and enhancing cellular uptake [61]. Nasal drug administration is promising for the treatment of neurodegenerative diseases, including Alzheimer’s disease (AD), due to the ability to bypass the blood-brain barrier (BBB), ease of application, and high patient compliance [62,63]. AD is the most prevalent neurological disorder affecting the elderly population. Currently, AD lacks a definitive cure, and the medications available provide only symptomatic relief. Several hypotheses have been proposed to explain the pathogenesis of AD, including the amyloid cascade hypothesis [64], tau hypothesis [65], hypothesis of mitochondrial dysfunction [66], the inflammation hypothesis [67], the glutamate hypothesis [68], etc. Contemporary approaches to the therapy of AD focus on the development of multifunctional compositions that can bind to several targets [45,69,70].

**Figure 1:**
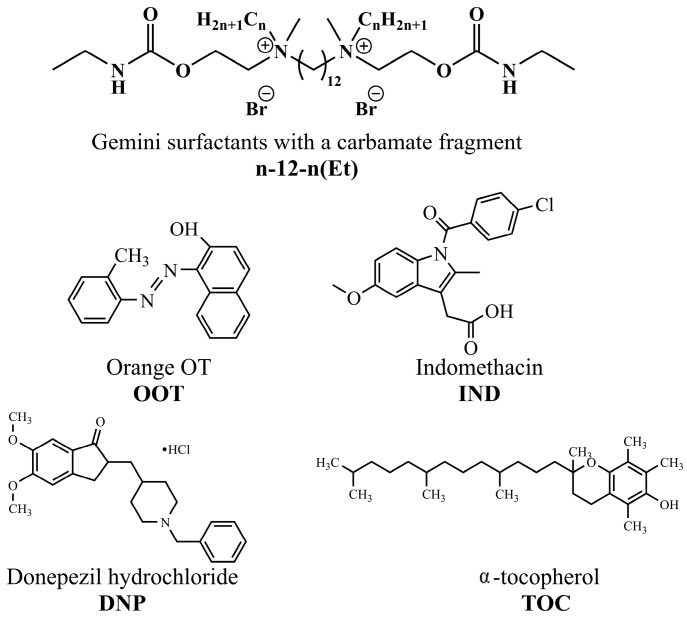
Structures of the compounds under study.

Previously, the approach of combined delivery of α-tocopherol (TOC) and donepezil hydrochloride (DNP) (Figure 1) in liposomes modified with tetradecyltriphenylphosphonium bromide was successfully tested in our research group [45]. In this work as a further step, to increase the bioavailability of the formulations, new GS with a dodecane spacer and biodegradable carbamate fragments were synthesized (Figure 1), which were then used to impart positive charge to liposomes. To achieve this goal, the following tasks were set: (1) to characterize the concentration thresholds for the aggregate formation by GS using a set of physical and chemical methods; (2) to evaluate the antimicrobial activity of new surfactants; (3) to optimize liposomal compositions during modification with GS; (4) to evaluate the ability of the modified liposomes to get in the brain; and (5) to test cationic liposomes for the therapy of transgenic mice with AD model (APP/PS1) in a behavioral memory test and histological analysis of amyloid beta (Aβ) plaques.

## 2. Results

**Figure 2:**
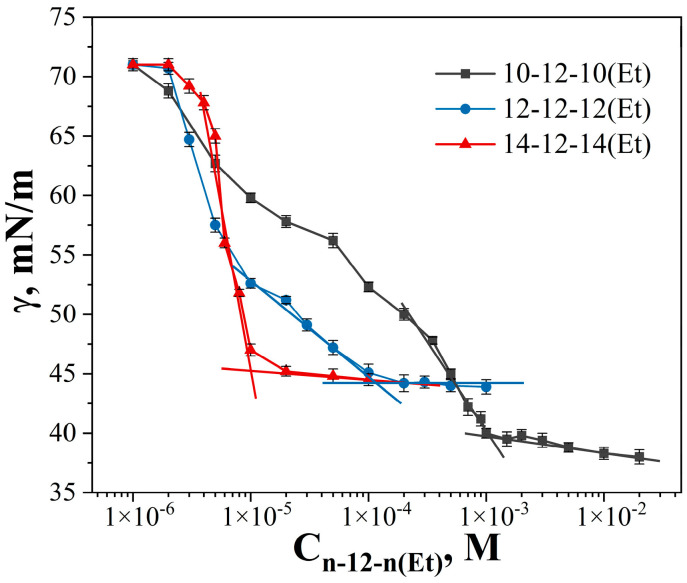
Surface tension isotherms of 10-12-10(Et), 12-12-12(Et), and 14-12-14(Et) aqueous solutions, 298 K.

**Figure 3:**
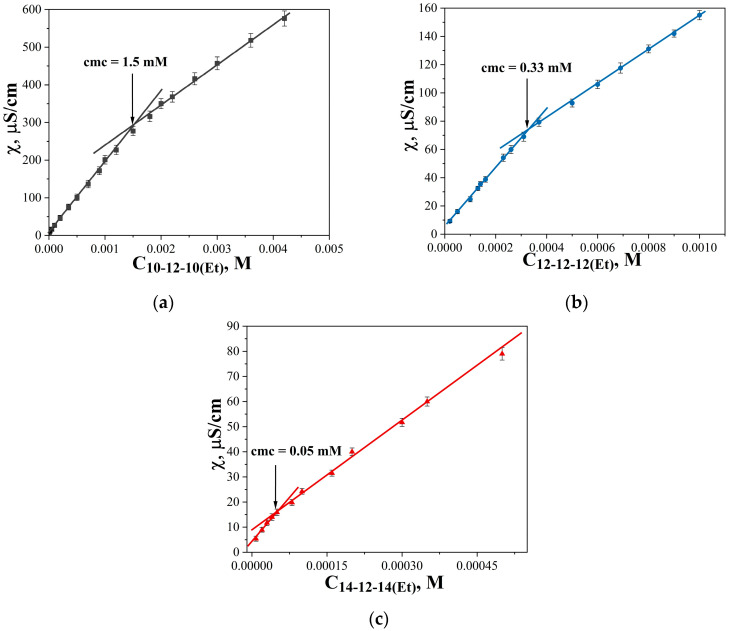
The dependence of the specific electrical conductivity of (**a**) 10-12-10(Et), (**b**) 12-12-12(Et), and (**c**) 14-12-14(Et) aqueous solutions on surfactant concentration, 298 K.

Dicationic surfactants with decyl, dodecyl, and tetradecyl hydrophobic tails and dodecane spacer (n-12-n(Et)) were successfully synthesized and characterized using ESI mass spectrometry, as well as ^1^H NMR and FTIR spectroscopy. A detailed description of the synthesis of each homologue and the spectra are given in the SI (Figures S1–S6). A plot of surface tension versus n-12-n(Et) concentration is shown in Figure 2: surface tension decreases with increasing surfactant concentration and comes to a plateau. The breakpoints of the dependence correspond to the cmc, which are summarized in Table 1. As expected, an increase in surfactant alkyl chain length leads to a decrease in the cmc values, since the hydrophobic effect is the driving force for micellization in aqueous solutions. For 10-12-10(Et) and 12-12-12(Et) a gentle slope of surface tension isotherm is observed, while for 14-12-14(Et) a sharper decrease in surface tension is clearly seen, with the cmc value occurring in the micromolar range. The cmc values were also obtained as the intersection of two linear segments of the conductivity dependence on surfactant concentration (Figure 3).

From the dependence of surface tension and conductivity on surfactant concentration, the thermodynamic parameters of adsorption and micellization were calculated (Table 1). The values of maximum surface excess (Г_max_) and surface area per surfactant molecule (A_min_) change nonmonotonically with an increase in the length of the hydrocarbon tails. Negative free energy of micellization (∆G_mic_) increases linearly in magnitude with an increase in the alkyl tail length by two methylene groups. The degree of counterion binding remains approximately the same for all homologues, with a slightly higher value for the tetradecyl homologue (0.44). The degree of counterion binding for 12-12-12(Et) and 14-12-14(Et) was also determined using a bromide selective electrode (Table 1, Figure S7). For the dodecyl homologue, the values obtained by potentiometry coincided with the data obtained from conductometry. Despite some differences obtained for the tetradecyl homologue, it can be concluded that GS have lower β values compared to monocationic surfactants.

**Figure 4:**
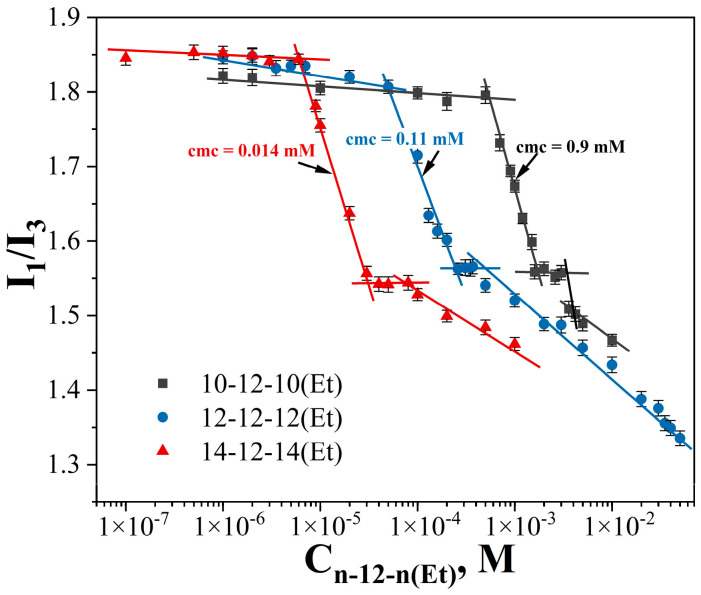
Ratio of the fluorescence intensity of the first (373 nm) and third (383 nm) peaks of pyrene in n-12-n(Et) aqueous solutions, 298 K.

The use of spectral probes to study of self-organization makes it possible to simultaneously determine several important parameters for the systems under study: cmc, polarity parameter, aggregation number, and solubilizing capacity. Based on the fluorescence spectra of pyrene (Figure S8), the dependence of the probe polarity parameter on the surfactant concentration was plotted (Figure 4). Plateaus at the polarity parameter values at the level of approximately 1.5 make it possible to determine the cmc values at the middle of the sigmoid curve (Table 2). It should be noted that such high values of the polarity parameter of pyrene solubilized into micelles of GS indicate a relatively polar microenvironment of the probe. After the dependence plateaus, a further decrease in the polarity parameter is observed, indicating a deeper penetration of the probe into the micellar core due to, for instance, a size increase.

**Figure 5:**
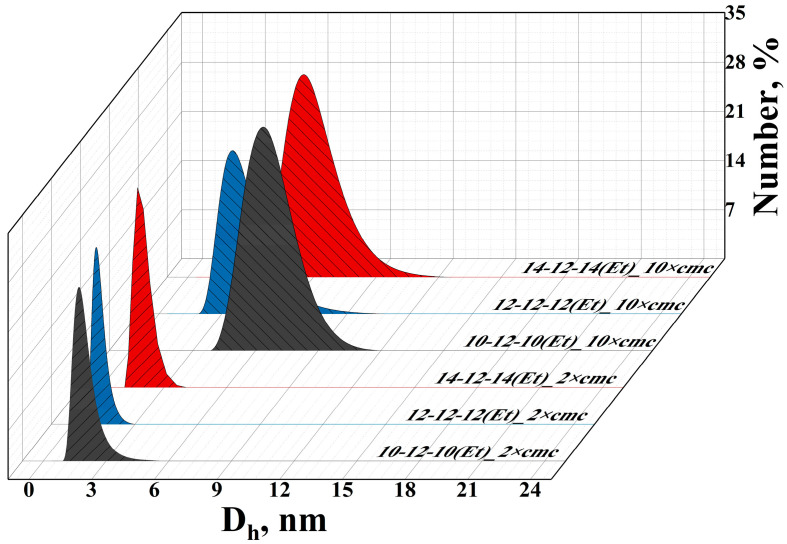
Number-averaged size distribution of n-12-n(Et) aggregates, 298 K.

The size of the n-12-n(Et) aggregates was assessed using dynamic light scattering (DLS) (Figure 5). The measurements were carried out in two surfactant concentration regimes: 2 times higher than the cmc values (2 × cmc) and 10 times higher than the cmc values (10 × cmc). As can be seen, for concentrations close to the cmc value, small micellar aggregates with a hydrodynamic diameter of 2 nm are formed. An increase in surfactant concentration to a region 10 times higher than the cmc leads to a 2-fold increase in the hydrodynamic diameter of aggregates up to 4–6 nm. The morphology of the n-12-n(Et) aggregates can be evaluated from the values of the steady-state fluorescence anisotropy (r) of 1,6-diphenyl-1,3,5-hexatriene (DPH). Usually, the fluorescence anisotropy of DPH is higher than 0.14 typical for bilayer aggregates and is lower than 0.14 characteristic for spherical or rodlike micelles [71]. This difference is due to the packing density of hydrocarbon chains in vesicular and micellar aggregates. For n-12-n(Et), r values of no more than 0.14 were established (Table S1), which, together with the small hydrodynamic diameter of the aggregates, allowed us to assume that micellar aggregates are formed in an aqueous solution.

**Figure 6:**
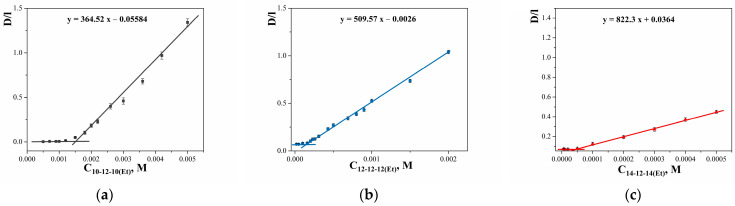
The dependence of the reduced optical density of saturated solutions of OOT at 495 nm on surfactant concentration for (**a**) 10-12-10(Et), (**b**) 12-12-12(Et), and (**c**) 14-12-14(Et), 298 K.

An important property of surfactants, which determines their wide practical application, is solubilization, i.e., colloidal dissolution of hydrophobic substances in micelles. The solubilization process was observed after reaching the cmc. To estimate the values of cmc and solubilizing capacity, a model hydrophobic dye Orange OT (OOT) with a convenient band in the visible part of the spectrum was used. Based on the absorption spectra of surfactant solutions containing an excess of the hydrophobic dye OOT (Figure S9), the dependence of optical density on the surfactant concentration was plotted (Figure 6). The slope after the cmc value, that is, the region of a sharp increase in optical density, allows us to calculate the solubilizing capacity (Table 2). These values doubled with an increase in the length of GS hydrocarbon tails.

**Figure 7:**
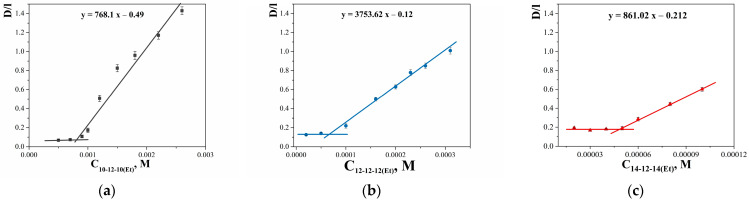
The dependence of the reduced optical density of saturated solutions of IND at the absorption maximum on surfactant concentration for (**a**) 10-12-10(Et), (**b**) 12-12-12(Et), and (**c**) 14-12-14(Et), 298 K. λ_max_ is equal to 324, 322, and 320 nm for 10-12-10(Et), 12-12-12(Et), and 14-12-14(Et), respectively.

To test the established tendency for the solubilization of hydrophobic substrates, similar studies were carried out for indomethacin (IND). For this, the values of the extinction coefficient of IND in an aqueous solution of each surfactant and the wavelength of maximum absorption were determined (Figure S10). The dependence of the reduced optical density of surfactant solutions with IND was linearized above the cmc to calculate the solubilizing capacity (Figure 7 and Figure S11, Table 2). The aggregation number of GS was calculated (Table 2) using the Schott approach [72] while making the following assumptions: (a) one dye molecule per micelle; (b) the concentration of surfactant not associated with micelles is constant and equal to the cmc. It was found that in the range 10-12-10(Et)—12-12-12(Et)—14-12-14(Et) the N_agg_ value decreases.

One of the important functional properties of cationic surfactants that may significantly widen their biomedical application is their antimicrobial effect. Therefore, GS were tested for antimicrobial activity toward Gram-positive and Gram-negative bacteria and fungi (Table 3). GS of the n-12-n(Et) series showed high antimicrobial activity comparable with the antibiotic ciprofloxacin and the antifungal drug ketoconazole. The minimum inhibitory concentration (MIC) values of carbamate-containing GS are clearly dependent on the length of the hydrocarbon tails and take a maximum for 10-12-10(Et). Ciprofloxacin is two orders of magnitude less effective toward the resistant strain MRSA-1 than 10-12-10(Et) [21]. Data on antimicrobial activity allow us to draw conclusions on the toxicity of the studied compounds toward microorganisms. Among GS, 14-12-14(Et) is the least toxic. The decyl homologue is promising for the creation of antimicrobial formulations, especially for resistant strains.

**Figure 8:**
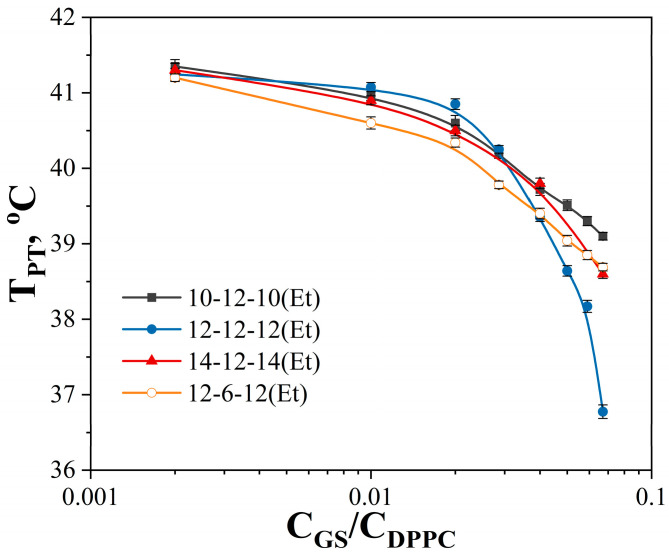
DPPC phase transition temperature versus surfactant/lipid molar ratio.

The fundamental characteristic of cationic surfactants, which determines their antimicrobial activity, namely, their ability to integrate into lipid membranes, can be assessed using model systems. The results of turbidimetric dependencies (Figure S12) processing on the incorporation of surfactants into the lipid bilayer based on 1,2-dipalmitoyl-sn-glycero-3-phosphocholine (DPPC) are presented in Figure 8, with 12-6-12(Et) analogue given for comparison. As can be seen, all GS result in a decrease in phase transition temperature (T_PT_) value, indicating the disturbance in the lipid bilayer and thereby testifying to the integration of amphiphilic molecules between the lipids. It is interesting to note that 12-12-12(Et) reduces the DPPC phase transition temperature more strongly compared with other GS, whereas 12-6-12(Et) demonstrates the same effect as 10-12-10(Et) and 14-12-14(Et). It can be assumed that the 12-12-12(Et) molecule has a more extended and mobile conformation, so it can disrupt the bilayer more strongly. Information about the ratio of surfactant/lipid causing the destruction of the lipid bilayer will be useful when choosing surfactant concentration in the preparation of modified liposomes.

In the next stage, the GS were tested as modifying agents for liposomes in the therapy of AD. The main composition of the liposomes was soy PC and Chol with a molar ratio of 3:2, and at a total concentration of 5, 10, and 15 mM. Several ratios of surfactant/lipid, namely 1/100, 1/50, 1/35, and 1/25, were selected for preparation of cationic liposomes. All liposome samples were prepared in HEPES buffer at pH 7.4. First, using 14-12-14(Et) as an example, the optimization of PC/Chol concentration was carried out. As observed in Table 4, the inclusion of 14-12-14(Et) led to a slight reduction in the hydrodynamic diameter of the liposomes, regardless of the lipid concentration. It is also worth noting that systems with different lipid concentration exhibited similar changes in zeta potential upon modification with different GS concentrations. Within 5 months, the liposomes did not undergo destructive changes and remained stable, probably due to their high charge and the storage conditions (277 K). During storage, systems with a PC/Chol concentration of 15 mM showed higher zeta potential for all ratios, with the maximum observed at the GS/lipid ratio of 1/35. It is also worth noting that the zeta potential of unmodified liposomes became negative during storage (approximately −23 mV, −22 mV, and −36 mV in the case of liposomes with a lipid concentration of 5, 10, and 15 mM, respectively) (Table 4).

After selecting the optimal concentration of the lipid, the composition was further optimized by choosing the most suitable GS homologue and its concentration. The first step involved evaluation of the size, monodispersity, and zeta potential of n-12-n(Et) modified liposomes. As seen from the data presented in Table 5, all systems were characterized by PdI not exceeding 0.1. However, during storage, the polydispersity and size of the systems increased, reaching a maximum for liposomes modified with 12-12-12(Et). For liposomes modified with decyl and tetradecyl homologues, size and PdI remained within 120–130 nm and 0.1–0.15, respectively, indicating high colloidal stability of the liposomes within 5 months.

**Figure 9:**
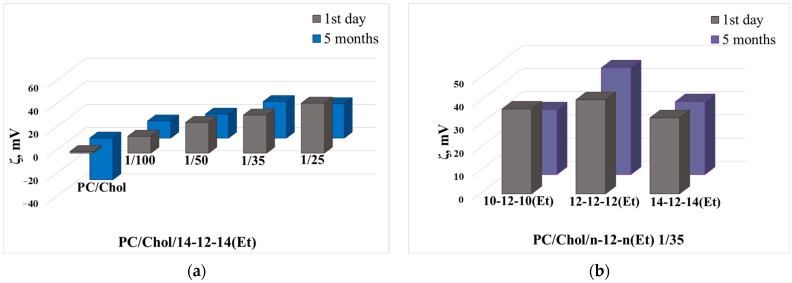
Zeta potential of (**a**) PC/Chol/14-12-14(Et) liposomes at various molar ratio of components and (**b**) PC/Chol/n-12-n(Et) liposomes at molar ratio of 1/35 on the 1st day and 5th month of storage.

Regarding the zeta potential of liposomes, the incorporation of GS into liposomes at a concentration 100 times lower than the lipid component led to an increase in the zeta potential of liposomes from +1.3 mV to +14–17 mV, while the addition of 1/25 of carbamate GS resulted in a further increase in zeta potential up to +43–48 mV, depending on the selected GS (Table 5). Diagrams illustrating the change in zeta potential of liposomes as the content of 14-12-14(Et) increases are shown in Figure 9a. It can be observed that within 5 months, the zeta potential of PC/Chol/14-12-14(Et) liposomes changed insignificantly. Additionally, it is worth noting that liposomes modified with the dodecyl homologue exhibited a higher zeta potential compared with the other two homologues (Figure 9b).

Before loading TOC and DNP into liposomes, a model probe, Rhodamine B (RhB), was encapsulated into the nanoparticles. Our research group has previously demonstrated that a concentration of 0.5 mg/mL of RhB is optimal for loading liposomes [43]. As seen from the data presented in Table 6, the encapsulation efficiency (EE) of RhB slightly increases with an increase in the number of methylene groups in the surfactant hydrocarbon tails. A more pronounced difference in the EE of RhB is observed when the concentration of PC/Chol is increased from 5 to 15 mM, providing compelling evidence in favor of choosing a system with a lipid content of 15 mM. In the case of TOC, EE for all systems was above 90%. It is also worth noting that encapsulation of TOC contributes to an increase in the hydrodynamic diameter of the liposomes, which increases with PC/Chol concentration. Such an effect was observed only in the case of loading the hydrophobic substrate TOC, whereas the size of liposomes remained unchanged during the loading of hydrophilic substrates RhB and DNP. The encapsulation of all substrates into liposomes has a minimal effect on their zeta potential (Table 6).

**Figure 10:**
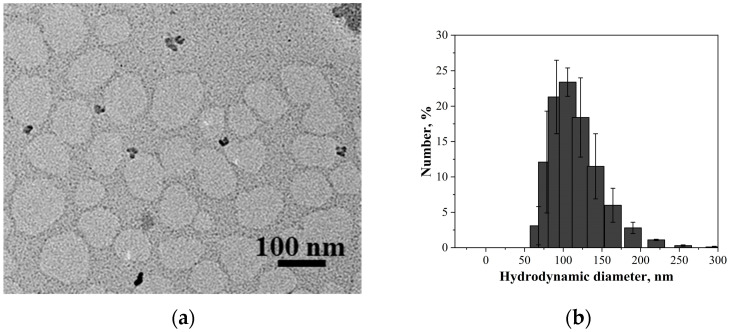
(**a**) TEM image and (**b**) number-averaged size distribution of particles (DLS) of PC/Chol/TOC/14-12-14(Et) liposomes (15 mM, 1/35), 298 K.

As an example, PC/Chol/TOC/14-12-14(Et) liposomes were visualized using transmission electron microscopy (TEM) (Figure 10a). Aggregates with a diameter of 100 nm are observed in the micrographs. It should be noted that the system exhibits a certain polydispersity, and aggregates smaller or larger than 100 nm are also observed. This result is expected, since the system was also analyzed using DLS prior to microscopic imaging (Figure 10b). From the presented diagram, it can be seen that the solution initially contained aggregates with a size distribution ranging from ≈ 70 to ≈ 160 nm with a diameter predominance of 100 nm.

**Figure 11:**
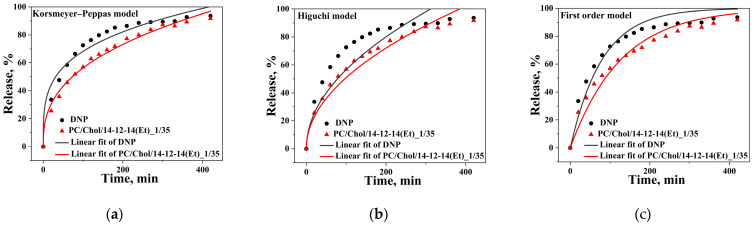
The release kinetic model fitting curves of free DNP and DNP encapsulated in PC/Chol/14-12-14(Et): (**a**) Korsmeyer–Peppas model; (**b**) Higuchi model; (**c**) first-order kinetic model. The total lipid concentration is 15 mM, surfactant/lipid molar ratio is 1/35. Phosphate buffer (0.025 M), pH = 7.4, 310 K.

Monitoring of the release rate of DNP from liposomes was conducted in vitro using PC/Chol/14-12-14(Et) system (15 mM, 1/35) (Figure 11). A free DNP was chosen as a comparative system. It was found that the inclusion of DNP in the modified liposomes reduced its release rate from the dialysis bag. It is worth noting that the release rate of DNP is independent of the surfactant concentration in the lipid bilayer and slows down almost equally (Figure S13). Additional analysis tools for the DNP release curves included mathematical equations (models) describing the release kinetics, namely the Korsmeyer-Peppas (Figure 11a), Higuchi (Figure 11b), and first-order models (Figure 11c). Visually, it can be seen that the Korsmeyer-Peppas model better fits the release curves of free and liposomal DNP compared with the Higuchi and first-order models. A similar trend was also observed for liposomes with different concentration of 14-12-14(Et) in the bilayer (Figure S13). Confirmation of this is also evident from the correlation coefficients (R^2^), which exceed 0.99 for the liposomal DNP (Table S2). For the Higuchi and first-order models, R^2^ values are ≤97. It is worth noting that for free DNP, the R^2^ values are noticeably lower in all cases (0.96, 0.75, and 0.94 for the Korsmeyer-Peppas, Higuchi, and first-order models, respectively). According to the calculated rate constants (k) within each model, the inclusion of DNP in liposomes reduces its release rate from the dialysis bag (Table S2), as mentioned above. The absorption spectra of the DNP are presented in Figure S14.

Before in vivo experiments, GS were tested for their inhibitory effect on human acetylcholinesterase (hAChE) and butyrylcholinesterase (hBChE) in vitro. The half maximal inhibitory concentration (IC_50_) was evaluated. High activity in the nanomolar range was observed for all homologues toward hAChE. The best inhibitory activity was shown for 14-12-14(Et) with the IC_50_ value of 11.0 ± 0.5 nM. It was lower than that for 10-12-10(Et) (43.1 ± 0.9 nM) or 12-12-12(Et) (82.1 ± 13 nM) (Table 7). Interestingly, the tetradecyl homologue inhibited hBChE two orders of magnitude less effectively than hAChE (1.44 ± 0.5 μM) indicating selectivity of 14-12-14(Et) toward hAChE.

**Figure 12:**
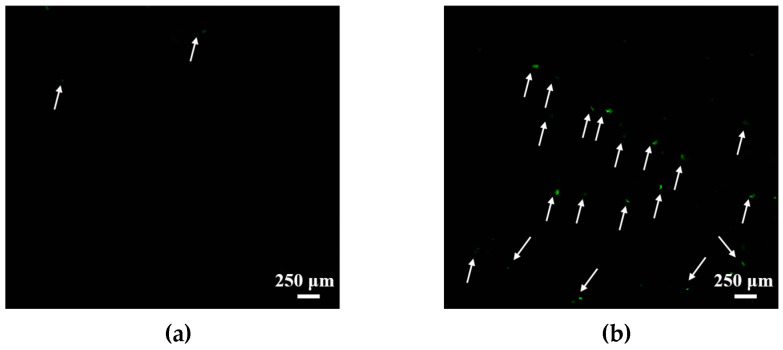
Cross-sections of rat brain: (**a**) after administration of free RhB (0.5 mg/kg); (**b**) after intranasal administration of RhB (0.5 mg/kg) in PC/Chol/TOC/14-12-14(Et) (10 mM) liposomes. Scale bar 250 μm. Arrows indicate RhB fluorescence.

The first stage of in vivo experiments involving laboratory animals was evaluation of liposome (PC/Chol/TOC/14-12-14(Et)) penetration into the rat brain after intranasal administration. For this purpose, the liposomes were loaded with the fluorescent dye RhB. A water solution of free RhB was used as control. As a result, significantly higher green fluorescence of RhB was observed after intranasal administration of the liposomal RhB compared with the free dye (Figure 12).

**Figure 13:**
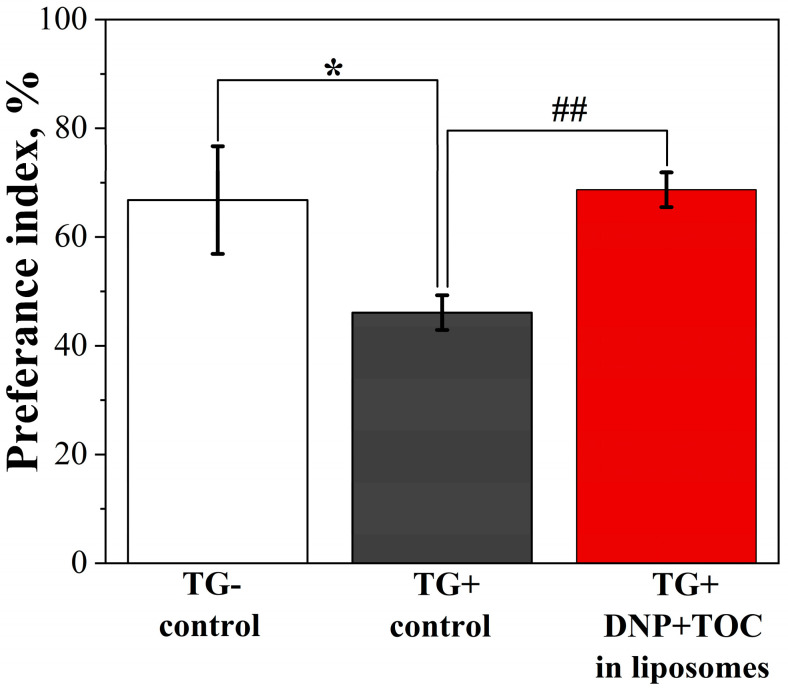
Distribution of the preference index of transgenic APP/PS1 mice for the novel object in the control group of wild-type mice (TG−), in the control group of transgenic mice (TG+), and in the group of transgenic mice (TG+) that intranasally received liposomes with TOC and DNP for 21 days. Data are presented as mean values ± SEM. *—The difference with regard to the control group of TG− mice is statistically significant at *p* ≤ 0.05; ##—the difference with regard to the control group of TG+ mice is statistically significant at *p* ≤ 0.01. Statistical analysis was performed using the Mann–Whitney test.

After all stages of system optimization and characterization, both in vitro and in vivo, the leading system was tested as a drug delivery form for the therapy of transgenic mice with an AD model. For this, PC/Chol/14-12-14(Et) liposomes (15 mM, 1/35) loaded with TOC and DNP were intranasally administered to mice for 21 days. Initially, memory parameters in the mice were assessed using the “novel object recognition” behavioral test. Upon memory restoration during therapy, the mice showed a greater preference for exploring novel objects. In the case of the control group of transgenic mice (TG+), the preference for the novel object was significantly lower (46.1 ± 3.2%, *p* = 0.047) compared with the control group of wild-type mice (TG−) (66.8 ± 9.9%). Conversely, the group of transgenic mice that were intranasally injected with liposomes with DNP and TOC showed an increased interest in novel objects with a probability of 68.7 ± 4.98%, which was significantly higher (*p* = 0.002) than in the TG+ control group, indicating the restoration of memory deficit (Figure 13).

**Figure 14:**
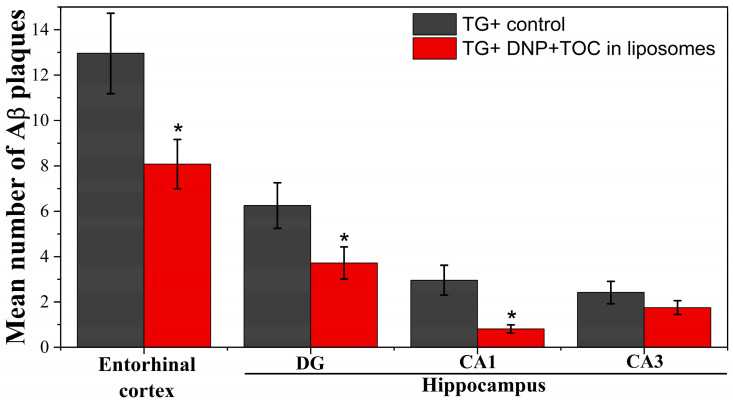
Mean number of Aβ plaques in the entorhinal cortex and hippocampus of the brain in the control group of transgenic mice (TG+) and in the group of transgenic mice (TG+) that received liposomes loaded with TOC and DNP. Data are presented as mean values ± SEM. *—The difference with regard to the TG+ control group is statistically significant at *p* ≤ 0.05. Statistical analysis was performed using the Mann–Whitney test.

The accumulation of Aβ plaques in the brain is one of the major pathological phenomena of AD, associated with the death of neurons. Therefore, the effectiveness of liposomal therapy was determined by calculating the number of Aβ plaques in the brains of APP/PS1 transgenic mice using Thioflavin S (ThS) staining. The results of this study showed that intranasal administration of liposomes loaded with TOC and DNP reduced in the mean number of Aβ plaques in the hippocampus and entorhinal cortex of the mouse brain (Figure 14). Specifically, in the entorhinal cortex of the mice brain, the mean number of Aβ plaques decreased from 12.96 ± 1.78 to 8.08 ± 1.01, in the DG area from 6.25 ± 1.01 to 3.72 ± 0.71, and in the CA1 area from 2.96 ± 0.66 to 0.81 ± 0.18. The reduction was statistically significant (*p* ≤ 0.05), indicating the potential of the proposed liposomal drug formulation in AD therapy.

## 3. Discussion

Tensiometry and conductometry are two classical and informative methods for investigating new surfactant properties at the air/water interface and in the bulk solution. In addition to the cmc values, these methods allow us to determine thermodynamic parameters (Table 1). Generally, the presence of two hydrocarbon tails in one GS molecule leads to very low cmc values compared with monocationic analogues [73,74]. It is known that the dependence of the cmc of n-s-n type GS on the length of the spacer fragment has a maximum value at s = 6 [75,76]. In comparison with the cmc values of the previously studied GS of the n-6-n(Et) type (5.8, 0.5, and 0.03 mM for 10-6-10(Et), 12-6-12(Et), 14-6-14(Et), respectively) [21], new amphiphiles with dodecane spacer n-12-n(Et) have lower cmc values (1, 0.11, and 0.013 mM for 10-12-10(Et), 12-12-12(Et), 14-12-14(Et), respectively) (Table 1). This may be due to the fact that the flexible long hydrocarbon spacer can fold and contribute to the enhancement of the hydrophobic effect during micelle formation.

The value of Γ_max_ decreases and the value of A_min_ increases as the length of the hydrocarbon chain increases from C_10_ to C_12_, but for the tetradecyl homologue, a sharp change in these parameters to the opposite trend is observed. Similar non-monotonous dependences were previously observed for GS with isopropyl and 2-hydroxyethyl fragments in the head group [73,77]. The molecule of GS studied contains a carbamate fragment capable of forming additional bonds. Based on the calculated adsorption layer thickness (0.7, 0.44, and 3.3 nm for 10-12-10(Et), 12-12-12(Et), 14-12-14(Et), respectively), it was assumed that in the case of tetradecyl homologue, the thickness of the surfactant layer at the air/water interface is more than a monolayer, which explains the high values of maximum surface excess. The relatively low values of the degree of binding of the bromide counterion for GS with carbamate fragments and a dodecane spacer can be explained, firstly, by the relatively low charge density in the case of spatially separated ammonium groups [78,79], and secondly, by charge shielding by additional substituents in the head group [80].

Fluorescence spectroscopy is another way to determine the cmc values. This method is widely used to study surfactant properties due to its high sensitivity and very low concentration of the injected probe [81]. By calculating the ratio between the intensity of the first peak (I_1_ = 373 nm) and the third peak (I_3_ = 384 nm) of pyrene, it was shown that the polarity parameter for GS studied on the plateau corresponds to I_1_/I_3_ in 2-Pentanone [82]. Even the presence of a long hydrophobic spacer does not reduce the polarity parameter in comparison with carbamate-containing surfactants of n-6-n(Et) type [21]. The determination of the cmc was carried out at the middle of the sigmoidal curve according to the published method [83]. For n-12-n(Et) amphiphiles, the micelle formation thresholds obtained by fluorimetry are in good agreement with the conductometry and tensiometry data (Table 1 and Table 2).

The size and morphology behavior of n-12-n(Et) micellar aggregates were examined by DLS (Figure 5) and steady-state fluorescence methods. The obtained values of the DPH fluorescence anisotropy (Table S1) for n-12-n(Et) are in full agreement with the previously obtained values for another GS, namely alkanediyl-α,ω-bis(dimethyldodecylammonium bromide) [84].

The solubilizing capacity (S) values for the GS studied are given in Table 2. It was found that the S values for surfactants with a dodecane spacer are higher than for n-6-n(Et) type GS: 0.015, 0.024, 0.032 mol_OOT_/mol_GS_ for 10-6-10(Et), 12-6-12(Et), 14-6-14(Et), respectively [21] and 0.021, 0.029, 0.047 mol_OOT_/mol_GS_ for 10-12-10(Et), 12-12-12(Et), 14-12-14(Et), respectively (Table 2). Moreover, this difference is most clearly observed for tetradecyl homologue, which may indicate a favorable orientation of the dodecane spacer, providing a 1.5-fold increase in the 14-12-14(Et) solubilizing capacity. The values obtained for OOT exceed the values for alkylammonium analogues [85], which allows us to increase the functional activity several times at a significantly lower concentration of surfactants. The obtained regularity is confirmed by the example of IND solubilization in the n-12-n(Et) micellar solution (Table 2).

Another difference between the series of surfactants under study and classical amphiphiles is the trend in the N_agg_ change with an increase in the surfactant alkyl tail length. For example, for a series of alkyltrimethylammonium bromides, the N_agg_ values are 55 (C_12_TAB), 70 (C_14_TAB), and 89 (C_16_TAB) [86]; for pyrrolidinium bromides (L-C_n_PB) N_agg_ values are 42 (L-C_12_PB), 48 (L-C_14_PB), and 53 (L-C_16_PB) [87]. That is, an increase in the hydrophobicity of classical monocationic surfactants leads to an increase in N_agg_. In our case, the trend is reverse: the N_agg_ values calculated by the Schott approach are equal to 50, 28, and 16 for 10-12-10(Et), 12-12-12(Et), and 14-12-14(Et), respectively (Table 2). This trend was observed earlier for other GS. For instance, the N_agg_ values determined using pyrene fluorescence quenching for cationic GS based on the adamantane spacer [C_12_E-Ad-EC_12_], [C_14_E-Ad-EC_14_], and [C_16_E-Ad-EC_16_] vary in the order of 15, 11, and 6, respectively [88]. It is known that elongation of the spacer fragment leads to a decrease in N_agg_ values [89]. This may be due to additional conformational freedom that allows alkyl chains to pack into more compact aggregates. This pattern was also confirmed for our compounds; for instance, the N_agg_ values were 29 for 14-6-14(Et) and 16 for 14-12-14(Et).

As a rule, Gram-negative bacteria are more resistant to the action of foreign membranotropic compounds, including surfactants, which are associated with the two-layer structure of their membrane. There is a point of view that the antimicrobial activity of surfactants is associated with their ability to disrupt the integrity of the cell membrane [90]. However, recent studies on the antimicrobial activity of mono- and dicationic imidazolium surfactants using fluorescent labels have shown that the mechanism of leading compound action is specific and is not associated with the destruction of the membrane [91,92]. For n-12-n(Et), the antimicrobial activity also decreases with an increase in the alkyl tail length (Table 3), as was previously shown for both dicationic imidazolium surfactants [92] and for n-6-n(Et) type carbamate-containing surfactants [21].

For 14-6-14(Et), hemolytic activity was evaluated in terms of HC_50_ (concentration of the test compound that causes 50% erythrocyte hemolysis), which is equal to 14 µg/mL. This value is higher than the HC_50_ value for the classic cationic surfactant cetyltrimethylammonium bromide [92] and reference drug Gramicidin S [93]. Therefore, the GS with carbamate fragments is less toxic. It should be additionally emphasized that only a small fraction of the GS was used for the modification of liposomes, which minimized the hemolytic activity of the formulation even more.

The properties of phospholipid membranes are largely determined by the phase transition temperature (T_PT_), which characterizes the structural transition of a lipid from a disordered liquid crystalline phase to an ordered gel phase [94]. In particular, a decrease in T_PT_ value indicates a perturbation of the lipid organization, for example, due to the incorporation of foreign molecules [95]. In this work, turbidimetry was used to investigate the incorporation of n-12-n(Et) into the lipid bilayer of DPPC. For the DPPC bilayer, the T_PT_ is equal to 41.4 ± 0.1 °C. Figure 8 shows the dependence of T_PT_ on the surfactant/DPPC molar ratio. All surfactants decrease the T_PT_ values up to a certain critical molar ratio, after which the solubilization of liposomes occurs. As can be seen, marked differences are observed in the behavior of 12-12-12(Et) and 12-6-12(Et), which is consistent with the literature data on the effect of the spacer fragment length on T_PT_ [18].

To sum up the results on self-organization of new n-12-n(Et) type dicationic surfactants, the following key patterns can be identified: (1) in the series 10-12-10(Et), 12-12-12(Et), 14-12-14(Et), the tetradecyl homologue has the lowest cmc values, the highest solubilizing capacity, the lowest toxicity, and the optimal ability to integrate into the lipid bilayer while maintaining its integrity; (2) comparison of all parameters studied between two series of surfactants, i.e., n-12-n(Et) and n-6-n(Et), differing only in the length of the spacer fragment, made it possible to establish the advantage of carbamate surfactants with a dodecane spacer.

After comprehensively studying the physicochemical characteristics of the n-12-n(Et) surfactants, they were tested as a modifying agent for liposomes. The practice of incorporating cationic surfactants into the lipid bilayer of liposomes remains a relevant direction, as it allows for increasing the zeta potential of aggregates and, consequently, influencing their colloidal stability [35,59,96,97]. It has also been demonstrated that cationic components (including surfactants) have an affinity for cell membranes [98], organelles [99,100], and bacterial cell membranes [5]. In addition, cationic liposomes can utilize adsorption-mediated transcytosis, which is widely recognized as the main BBB entry mechanism for cationic proteins and nanocarriers [48,63,101,102]. Previously, various GS have already been successfully incorporated into the lipid bilayer [42,103,104], including those with carbamate [21] and hydroxyethyl [56] fragments and different spacer length. In this study, liposomes based on soy PC and Chol, modified with n-12-n(Et), were obtained to load the antioxidant TOC and the acetylcholinesterase inhibitor DNP for AD therapy. In the initial stage, the influence of lipid concentration on liposome hydrodynamic diameter and zeta potential was assessed, since it was already shown that lipid concentration can directly affect the physicochemical parameters of liposomes [45]. DLS, which is the most common method for determining the size of liposomes, showed that the obtained modified nanoparticles had a size of approximately 110 nm, regardless of the lipid content or the surfactant/lipid ratio (Table 4 and Table 5). Most commercially available liposomal formulations have a unilamellar structure with a size of around 100 nm, which allows them to circulate in the bloodstream longer and reach targets more effectively [26].

An increase in the proportion of dicationic surfactants in the bilayer led to an increase in the liposome zeta potential (Table 5, Figure 9a), while the GS hydrocarbon tail length had a minor impact on the zeta potential (Table 5). It is worth noting that liposomes modified with 12-12-12(Et) exhibited the highest zeta potential (Figure 9b), but at the same time, these systems showed poorer stability (Table 5). This is likely due to the spatial arrangement of the surfactant in the lipid bilayer. Previously, other authors have shown that the ability of surfactants to integrate into the lipid bilayer depends on the length of the spacer fragment; specifically, surfactants with spacer lengths of 12, 14, and 20 methylene groups are incorporated better into liposomes (by having polar head groups located on opposite sides of the membrane layers) than those with spacers composed of 4 and 6 methylene units, which was also confirmed in the current study (Figure 8) [55]. Probably, in this case, 12-12-12(Et) serves as an intermediate member in the GS series, which, due to its structure, introduces a destabilizing rather than stabilizing effect on the lipid bilayer. This could also be related to the structure of the PC bearing 15 and 17 carbon atoms and unsaturated bonds in hydrophobic tails, which also contribute to the arrangement of surfactant molecules within the lipid bilayer.

In the next stage, liposomes modified with n-12-n(Et) at a surfactant/lipid ratio of 1/35 were loaded with several substrates, namely, RhB, TOC, and DNP. Substrate loading had a minimal effect on the physicochemical properties of the liposomes, except for TOC encapsulation. It was demonstrated that 10% TOC slightly increased the size of the liposomes to 122-142 nm depending on the modifying agent (Table 6). A similar influence of TOC on DLS data was previously shown by our research group [45]. For all substrates, high EE values were observed with a slight dependence on the hydrocarbon tail length (10-12-10(Et) < 12-12-12(Et) < 14-12-14(Et)) and the concentration of the lipid component (5 < 10 < 15 mM). The morphology of the obtained liposomes was further confirmed using TEM (Figure 10a), and the results were in good agreement with the DLS data (Figure 10b).

One of the key characteristics of liposomal systems is the rate of drug release from nanoparticles. The experimental data on the release kinetics of DNP were compared to three release models: Korsmeyer-Peppas, Higuchi, and first-order kinetic models. As shown in Figure 11, liposomal DNP exhibited a more prolonged release in the initial part compared with its free form, indicating a positive trend toward reducing systemic drug toxicity by providing a gradual release into the bloodstream. This observation is further supported by the rate constant values (k) (Table S2). It is worth noting that the 14-12-14(Et)/lipid molar ratio does not significantly affect the drug release rate (Figure S13). From the values presented in Table S2, it becomes evident that the Korsmeyer-Peppas model is the most suitable for describing the DNP release kinetics due to the highest correlation coefficient (R^2^) (≥0.99). It was also demonstrated that the release of DNP follows Fickian diffusion, since the diffusion release exponent (n) values are within the range of 0–0.45 [105]. It is worth noting that the Korsmeyer-Peppas model has been successfully applied in previous studies to describe the release kinetics of various substrates from liposomal systems [106,107,108], including DNP [109].

It is known that one of the types of symptomatic AD therapy is the inhibition of the cholinesterase enzyme [110]. In addition to the approved acetylcholinesterase inhibitor DNP (reversible inhibitor), carbamates (pseudo-irreversible inhibitors), e.g., rivastigmine, have gained significant attention [111,112]. Since the investigated GS contain a carbamate fragments in their structure, it was of interest to test their inhibitory activity toward hAChE and hBChE in vitro, with the hope that the combined action of carbamate-containing GS and DNP would contribute to slowing down AD progression. As evident from the data presented in Table 7, almost all homologues exhibit inhibitory activity in the nanomolar range toward both hAChE and hBChE. Of particular interest is 14-12-14(Et), as it shows the lowest IC_50_ values (against hAChE), almost 4 and 7 times lower compared with the decyl and dodecyl homologues. Additionally, 14-12-14(Et) also demonstrates significant selectivity toward hAChE (SI = 131), while in the case of decyl (SI = 0.44) and dodecyl (SI = 0.74) homologues, significant selectivity could not be achieved, suggesting that the alkyl chain length of the surfactant plays a key role in inhibiting both hAChE and hBChE, as demonstrated by other authors too [113].

The BBB is a well-known obstacle that contributes to the problem of low efficiency of most novel medicines in the treatment of the central neural system (CNS). It is considered to be represented by a neurovascular unit surrounding the cerebral blood vessels, which consists of endothelial cells, microglia, astrocytes, pericytes, neurons, and extracellular matrix [114]. The endothelial cells function as the main barrier due to the presence of enzymes and tight junctions and act as highly selective filters for all external compounds [102]. Even after bypassing multiple obstacles, most drugs are subject to efflux by ATP-binding cassette transporters [115], which makes the intranasal route of administration very attractive, as it allows the BBB to be bypassed, delivering the drug directly to the CNS [116]. In addition, the part of the drug formulation that does not directly enter the brain through the nasal route can enter the systemic bloodstream. This highlights the significance of drug nanoformulation as a highly promising strategy for improving drug bioavailability in the CNS [117]. The use of lipid carriers, such as solid lipid nanoparticles or liposomes, can improve the transport of drugs through the BBB by protecting them from efflux and providing sustained release [118,119,120]. Therefore, in the next stage, the ability of PC/Chol/TOC/14-12-14(Et) liposomes to reach the rat brain was evaluated using the fluorescent probe RhB. The brain section images clearly demonstrate that the fluorescence intensity of the liposomal form of RhB correlating with its ability to reach the brain is significantly higher compared with the free form of the probe (Figure 12). This result can be explained in terms of the liposome zeta potential. By incorporating GS into the lipid bilayer, the surface of the liposomes undergoes a charge reversal from negative to positive value, as demonstrated previously. This enables the liposomes to electrostatically interact with the negatively charged mucin in the nasal cavity, effectively “adhering” to it and remaining longer in the nasal cavity and reaching the brain [121].

The above findings provided the basis for further investigations involving the evaluation of the cognitive functions of mice with an AD model and quantification of Aβ plaques after liposomal therapy. The AD therapy was conducted for 21 days through intranasal administration of PC/Chol/14-12-14(Et) liposomes (15 mM, 1/35) loaded with TOC (1.5 mM) and DNP (0.5 mg/mL). To confirm the restoration of cognitive functions, a “novel object recognition” behavioral test was performed, which is widely used in experimental studies on memory impairment due to head trauma, aging, or neurodegenerative diseases [122]. According to the results obtained, the administration of liposomal TOC and DNP significantly increased the preference index in transgenic mice by approximately 22.6% compared with untreated mice with an AD model (Figure 13). Thus, the preference index for the mice receiving the liposomal drug (68.7 ± 4.98%) reached the level of healthy mice (TG-) (66.8 ± 9.9%). It is worth noting that in our previous study, the preference index increased only by ≈14%, which did not differ statistically from the control group of transgenic mice [45]. Importantly, no side effects were observed during the 21-day intranasal administration of the liposomes. None of the mice showed any signs of behavioral changes or movement difficulties. Their eating and drinking habits remained normal.

After completing the behavioral test, histological studies were conducted to quantitatively evaluate the Aβ plaques upon ThS staining. Analysis was carried out in the entorhinal cortex and hippocampus (DG, CA1, and CA3 areas), as these are crucial brain areas associated with memory function [123]. According to the data obtained, intranasal administration of the liposomal dispersion containing TOC and DNP to the mice significantly reduced the number of Aβ plaques by 37.6%, 40.5%, and 72.6% in the entorhinal cortex, DG, and CA1 areas, respectively, compared with the untreated group of mice with an AD model (Figure 14).

In summary, based on the liposomal part of the study, the following conclusions can be drawn: (1) n-12-n(Et) surfactants were successfully incorporated into the lipid bilayer of PC/Chol liposomes, thereby increasing the zeta potential of liposomes and enhancing their colloidal stability up to 5 months; (2) among the investigated GS, 14-12-14(Et) was the most optimal for liposome modification; (3) the most favorable physicochemical characteristics of liposomes were achieved at a PC/Chol concentration of 15 mM and a GS/lipid ratio of 1/35, which allowed for encapsulation of over 90% of the tested drugs TOC and DNP; (4) using the leader system, PC/Chol/14-12-14(Et), it was demonstrated that the release of DNP from liposomes occurs through Fickian diffusion. Furthermore, the modified liposomes successfully reached the brain of laboratory animals, leading to improved cognitive functions and a reduction in the number of Aβ plaques in the entorhinal cortex and hippocampus of transgenic mice with an AD model.

## 4. Materials and Methods

### 4.1. Chemicals

Soybean L-phosphatidylcholine (PC, 95%) and 1,2-dipalmitoyl-sn-glycero-3-phosphocholine (DPPC, >99%) were purchased from Avanti Polar Lipids (Alabaster, AL, USA). Ethyl isocyanate (98%), indomethacin (IND, ≥99%), HEPES buffer (>99.5%), donepezil hydrochloride (DNP, ≥98%), cholesterol (Chol, ≥99%), α-tocopherol (TOC), thioflavin S, pyrene (≥99%), Orange OT (OOT, 75%), 5,5′-dithiobis-(2-nitrobenzoic acid) (≥98%), human acetylcholinesterase (hAChE), human butyrylcholinesterase (hBChE), acetylthiocholine, and butyrylthiocholine were purchased from Sigma-Aldrich (St. Louis, MO, USA). Rhodamine B (RhB), 1,4-diazabicyclo [2.2.2]octane (DABCO, 97%), 1,6-diphenyl-1,3,5-hexatriene (DPH, 98%) were purchased from Acros Organics (Morris Plains, NJ, USA). Sodium phosphate buffer (PBS) was purchased from UralChemInvest (Ufa, Russia). Precursors for the GS synthesis—hydroxyethylated GS (n-12-n(OH))—were synthesized according to the published method [124]. Chloroform and ethanol (HPLC) were purchased from JSC №1 BASE Chemical reagents (Staraya Kupavna, Russia). Commercially available solvents (acetonitrile (JSC EKOS-1, Staraya Kupavna, Russia), ethyl acetate (Component-Reaktiv, Moscow, Russia), acetone (LLC “Chlorenchima”, Naro-Fominsk, Russia)) were purified by standard procedures before use. Micellar and liposomal dispersions were prepared using ultrapure Milli-Q water purified by the Simplicity^®^ UV system (Millipore SAS, Molsheim, France).

### 4.2. Synthesis of GS with Carbamate Fragment

Ethyl isocyanate (0.32 mL, 4 mM) was added to a stirred solution of n-12-n(OH) (1 mM) and DABCO (0.05 g). The reaction mixture was stirred for 16 h at 333 K in 30 mL dry acetonitrile. The solvent was removed under vacuum (20 mm Hg), and the product was recrystallized from ethyl acetate/acetone. The precipitate was filtered and dried on a water bath (313 K) under vacuum (15 mm Hg). Data from elemental analysis, IR spectroscopy, ^1^H NMR spectroscopy, and mass spectrometry were used to confirm the structures of the compounds. ^1^H NMR spectra were recorded on a Bruker Avance NMR ^1^H spectrometer. Mass spectra with electrospray ionization (ESI) were obtained on a Bruker AmaZon X Ion Trap mass-spectrometer (Bruker GmbH, Mannheim, Germany), and the results were processed using DataAnalysis 4.0 SP4 software. Elemental analysis was carried out on a EuroEA3028-HT-OM CHNS analyzer (Eurovector SpA, Pavia, Italy), and the results were processed using Callidus 4.1 software. IR spectra were recorded on a Tensor 27 Bruker spectrometer (Bruker GmbH, Mannheim, Germany) in KBr pellets, and the results were processed using OPUS 7/2012 software.

### 4.3. Tensiometry

The surface tension isotherms of the micellar solutions were determined using the Du Nouy platinum ring detachment method on a K6 tensiometer (KRŰSS GmbH, Hamburg, Germany). For the experiment, surfactant solutions with a volume of 10 mL were prepared. The measurements were carried out strictly at 298 K in glass beakers, the diameter of which exceeded the diameter of the platinum ring. Surface tension measurements were carried out until stable values were obtained. After each measurement, the ring was degreased in ethanol and thoroughly dried. The cmc of the micellar solutions was determined from the breakpoint on the isotherm of surface tension versus surfactant concentration. The thermodynamic parameters of adsorption and micellization were calculated for all systems based on the obtained tensiometric curves. The equations used are presented in Table 8.

### 4.4. Conductometry

The electrical conductivity of the micellar solutions was determined on an InoLab Cond 7110 conductometer (WTW, Weilheim, Germany). For the experiment, surfactant solutions with a volume of 10 mL were prepared. The electrical conductivity of all solutions was recorded at 298 K, and the electrode was washed with purified water after each measurement. The cmc was determined from the breakpoint of the dependence of electrical conductivity on the concentration of surfactants.

### 4.5. Electrode Potential Measurement

The electrode potential measurements were performed for the Br^−^ counterion using the I-160MI laboratory ionomer (JSC Scientific and Production Association of the Measuring Equipment, Moscow, Russia). Br^−^ selective electrode (ELIS-131Br) and a reference electrode (ESr-10101) were used for measurement of the electromotive force (Δ*E*) of aqueous solution of surfactants (the concentration from 0.02 to 0.5 mM for 12-12-12(Et); from 0.002 to 0.05 mM for 14-12-14(Et)). To calibrate the ionomer, a series of KBr solutions were prepared in the concentration range of 0.1 mM to 100 mM. The degree of counterion binding to aggregates (β) can be calculated from the mass balance for the surfactant ion and the counterion at any total concentration C_t_ using the following expression according to [125]:

β = 0.5(2C_t_ − C_Br^−^_)/(C_t_ − cmc).

### 4.6. Fluorescence Spectroscopic Measurement

To determine cmc by fluorimetry, a hydrophobic probe pyrene was added to a series of surfactant solutions at a concentration of 1 μM. The solutions were kept for 30–40 min for complete solubilization of the probe. The fluorescent emission spectra of pyrene were recorded on an F-7100 fluorimeter (Hitachi, Tokyo, Japan) using 10 × 4 mm quartz cuvette (Hellma Analytics, Müllheim, Germany) with the following settings: λ_ex_ = 335 nm, λ_em_ = 350–600 nm, and scanning speed = 1200 nm/min. The cmc was calculated from the dependence of the pyrene polarity index (the ratio of the intensities of the first (373 nm) and third (383 nm) peaks) on the surfactant concentration [83].

The micelle morphology was determined from the fluorescence anisotropy values of DPH on a fluorimeter equipped with a polarizer. DPH was added at a concentration of 0.175 mM to the surfactant solutions. The study was carried out with the following settings: λ_ex_ = 344 nm, λ_em_ = 400–600 nm, and scanning speed = 1200 nm/min. The anisotropy values were determined automatically at 450 nm.

### 4.7. Spectrophotometry

The cmc and the solubilizing capacity (S) of surfactants were determined using the spectral azo dye OOT and the non-steroidal anti-inflammatory drug IND. To obtain a saturated solution, an excess of OOT and IND was added to 2 mL of a surfactant solution, stirred, and kept for 48 and 24 h, respectively [12,126]. After that, the solutions were filtered using Millex^®^ Syringe Filters (Millipore, Burlington, MA, USA) with a pore size of 450 nm, and the optical density was measured. The absorption spectra were recorded on a Specord 250 Plus spectrophotometer (Analytik Jena AG, Jena, Germany). Quartz cells with a thickness of 1 and 0.5 cm (Hellma Analytics, Müllheim, Germany) were used for measurements.

The solubilizing capacity (S) was determined using the following formula:

S = b/ε,

where b is D/l = f (C_surf_) dependency slope (above cmc), ε is the extinction coefficient (17,400 L/mol∙cm for OOT, and the extinction coefficients of IND are determined in GS solutions and presented in Figure S10).

The OOT solubilization method (Schott method) was used to calculate the micelle N_agg_. The N_agg_ values were calculated for each point on the curve of dependence of the OOT-reduced optical density on the surfactant concentration above the cmc according to the presented formula:

N_agg_ = ε − (C_surf_ − C_cmc_)/D,

where ε is the extinction coefficient of OOT, C_surf_ is the surfactant concentration above cmc, and D is the optical density.

### 4.8. Antimicrobial Activity

The antimicrobial activity of the compounds was tested on Gram-positive *Staphylococcus aureus* ATCC 6538P FDA 209P (Sa), *Bacillus cereus* ATCC 10702 NCTC 8035 (Bc), *Enterococcus faecalis* ATCC 29212 (Ef), and Gram-negative bacteria *Escherichia coli* ATCC 25922 (Ec), *Pseudomonas aeruginosa* ATCC 9027 (Pa), including methicillin-resistant strains of *S. aureus* MRSA-1 (resistant to fluoroquinolones and beta-lactams) and MRSA-2 (resistant to beta-lactams). Antifungal activity was studied against *Candida albicans* ATCC 10231 (Ca), *Trichophyton mentagrophytes* var. gypseum 1773 (Tm), and *Aspergillus niger* BKMF-1119 (An). A more detailed description of the experiment is published in [21].

### 4.9. Hemolysis

The hemolytic activity was determined by comparing the optical density of hemoglobin released into the solution at 100% hemolysis and the optical density after treatment of the erythrocyte mass with a 14-6-14(Et) aqueous solution. A 10% suspension of human erythrocytes was used as the object of investigation. A more detailed description of the experiment is published in [37].

### 4.10. Turbidimetry

The temperature of the main phase transition was determined by turbidimetry in a 1 cm quartz cuvette using the Specord 250 Plus spectrophotometer (Analytik Jena AG, Jena, Germany), equipped with a Peltier thermostatic cell holder. Aliquots of surfactant solutions of a certain concentration were added to a liposomal dispersion of DPPC (0.7 mM) and incubated at room temperature for 25 min. Turbidity of liposomal dispersion was measured at a wavelength of 350 nm in the temperature range from 35 °C to 45 °C, with a measurement step of 0.2 °C and a waiting time of 80 s. The temperature accuracy was equal to 0.1 °C. The turbidity traces were approximated by the Van’t-Hoff equation for a two-state model providing a half-transition temperature value.

### 4.11. Dynamic and Electrophoretic Light Scattering

The hydrodynamic diameter, polydispersity index, and zeta potential of liposomes (diluted to 2 mM) and micellar solutions were determined using a U-shaped zeta cuvette on a Malvern ZetaSizer Nano instrument (Malvern Instruments Ltd., Worcestershire, UK), equipped with a helium-neon laser with a wavelength of 633 nm, a power of 10 kW, and a light scattering angle of 173°. The hydrodynamic diameter and zeta potential were calculated using the Stokes-Einstein and Smoluchowski equations, respectively, as presented in [48].

### 4.12. Liposome Preparation

Liposomes were obtained using the lipid film hydration method according to the algorithm published in [34]. The total lipid concentration (PC/Chol) at a molar ratio of 3/2 was varied in the range of 5, 10, and 15 mM. Liposomes were modified by incorporating GS with a hydrocarbon tail length of 10, 12, and 14 into the lipid bilayer. To select the optimal liposome formulation, the surfactant/lipid ratio was varied over a wide range, namely 1/100, 1/50, 1/35 and 1/25. TOC was mixed with lipids and surfactants at the stage of lipid film formation. To encapsulate hydrophilic substrates, the lipid film was hydrated with an aqueous solution of DNP or RhB (0.5 mg/mL). To obtain empty liposomes, the lipid film was hydrated with HEPES buffer (25 mM, pH = 7.4). To obtain 100 nm liposomes, the liposomal dispersion was extruded through a polycarbonate membrane using a LiposoFast Basic extruder (Avestin, Ottawa, ON, Canada). Liposomes were stored at 277 K.

### 4.13. Transmission Electron Microscopy

Lipid nanoparticles were visualized using TEM on a Hitachi HT 7700 Exalens instrument (Hitachi, Tokyo, Japan). Nanoparticle dispersion for imaging was diluted to 5 μM and deposited on a copper grid (Ted Pella, Pella, IA, USA) with a 15–25 nm carbon-formvar support film. Then, the sample was dried at room temperature for 60 min. The analysis was carried out at an accelerating voltage of 100 kV.

### 4.14. Encapsulation Efficiency and Release Rate of Substrate

The encapsulation efficiency of hydrophilic substrates was determined by separating unencapsulated substrates from liposomes using centrifuge filters (Amicon^®^ Ultra-0.5) with a pore size of 100 kDa (Merck Millipore, Burlington, MA, USA), and 0.4 mL liposomal dispersion was centrifugated for 10 min at 10,000 rpm in an Eppendorf MiniSpin microcentrifuge (Eppendorf, Hamburg, Germany). For the hydrophobic substrate, the method of extraction of the unencapsulated substrate in ethanol was used [47,127]. The concentration of both substrates was determined spectrophotometrically on Specord 250 Plus (Analytik Jena AG, Jena, Germany) using a 0.2 cm quartz cuvette (Hellma Analytics, Müllheim, Germany). Encapsulation efficiency (EE) was calculated using the following equation:

$$EE=\frac{Total\;amount\;of\;substrate-Amount\;of\;free\;substrate}{Total\;amount\;of\;substrate}\times 100$$

The extinction coefficients of the studied substrates were experimentally determined earlier in our research group (for RhB (λ_555_)—94,000 L/mol∙cm [56], for DNP (λ_317_)—9840 L/mol∙cm [45], for TOC (λ_285_)—2720 L/mol∙cm [45]).

The release rate of substrates from liposomes was determined using dialysis bags with a pore size of 3 kDa (Scienova GmbH, Jena, Germany), in which 2 mL of the test system was placed. The substrate was released into 0.025 M PBS (pH = 7.4) with a volume of 50 mL at 310 K and a stirring speed of 200 rpm. The optical density of the substrates in the external environment was determined using 1 cm quartz cuvettes (Hellma Analytics, Müllheim, Germany). The results are presented as a cumulative release percentage versus release time. Release profiles were fitted to Korsmeyer–Peppas, Higuchi, and first-order models using OriginPro 8.5 software (OriginLab Corporation, Northampton, MA, USA) according to the equations presented in [45].

### 4.15. In Vitro Cholinesterase Inhibitory Activity

Stock solutions (0.01 M) of the compounds were dissolved in H_2_O. The inhibitory activity of compounds against hAChE and hBChE was measured using Ellman’s method [128]. Enzyme-catalyzed hydrolysis was carried out in 0.1 M PBS (pH = 8.0) containing 0.1 nM hAChE or hBChE, 0.1 mM 5,5′-dithiobis-(2-nitrobenzoic acid) and 1mM acetylthiocholine or butyrylthiocholine as substrates. The tested compounds were incubated with the enzyme for 5 min prior to the addition of the substrate. Analyses were performed at 298 K using a Shimadzu UV-1800 spectrophotometer (Shimadzu Co., Kyoto, Japan) at 412 nm. The substrate hydrolysis rate was measured within 2 min. A sample without inhibitor was used as a control (100% cholinesterase activity). A sample without substrate was used as a blank. The experiments were conducted in triplicate. The percentage of enzyme inhibition was determined using OriginPro 8.5 software (OriginLab Corporation, Northampton, MA, USA) by plotting a percentage of the inhibition versus compound concentration. IC_50_ values (inhibitor concentration required to inhibit enzyme activity by 50%) were determined by the Hill equation:

$$\frac{E}{E_{max}}=\frac{{\left[I\right]}^{n}}{{IC}_{50}^{n}+{\left[I\right]}^{n}},$$

where E is enzyme activity in the presence of the compound, and [I] is the compound concentration.

### 4.16. Animals

In vivo experiments were carried out in accordance with the Directive of the Council of the European Union 2010/63/EU. The protocol of the experiments was approved by the Animal Care and Use Committee of FRC Kazan Scientific Center of RAS (protocol No. 2 from 9 June 2022). Animals were kept in a well-ventilated room at 293–295 K in a 12 h light/dark cycle with 60–70% relative humidity. Wistar rats were purchased from the Laboratory Animal Breeding Facility (Branch of Shemyakin-Ovchinnikov Institute of Bioorganic Chemistry, Puschino, Moscow Region, Russia). Transgenic mice with Alzheimer’s disease (AD) models were purchased from the Institute of Physiologically Active Substances, Federal Research Center of Problem of Chemical Physics and Medicinal Chemistry RAS (Chernogolovka, Moscow region, Russia).

### 4.17. Histology Analysis of Liposome Penetration into the Brain

Free RhB and RhB encapsulated in PC/Chol/TOC/14-12-14(Et) liposomes (10 mM) were administered intranasally to Wistar rats at a dose of 0.5 mg/kg (400 μL per rat). Untreated animals were used as controls. The animals were anesthetized with isoflurane (Laboratorios Karizoo, Barcelona, Spain) 1 h after injection and transcardially perfused with 300 mL of cold PBS (pH = 7.4). The rat brains were removed and frozen in liquid nitrogen. The samples were stored at 193 K, and 24 h before the experiment were transferred to a freezer with a temperature of 253 K. The obtained samples were cut (section thickness = 10 µm) using a Tissue-Tek Cryo3 microtome (Sakura Finetek, Torrance, CA, USA). RhB fluorescence in the rat brain was observed on a Leica TSC SP5 MP confocal laser scanning microscope (Leica Microsystems, Wetzlar, Germany) with a Cyanine 3 filter at λ_ex_ = 550 nm and λ_em_ = 570 nm.

### 4.18. Novel Object Recognition Test

The experiments were carried out on transgenic mice with an AD model (line B6C3-Tg(APP695)85Dbo (APP/PS1)). In the study group, mice were intranasally injected with liposomes with TOC and DNP (1 mg/kg) for 21 days, and the control group was injected with an equivalent amount of water (50 μL/mouse). On the 19th day of therapy, a new object recognition test was performed. During the test, the animals were injected with liposomes 20 min before the start. The experiment with two objects for recognition was carried out in a square test arena with black walls (50 cm long, 50 cm wide, 38 cm high). The test is based on the fact that the animal prefers to spend more time exploring a new object than an old one, which is an indicator of recognition memory. At the end of the test, a preference index was calculated according to the following equation:

$$Preference\;index=\frac{Exploration\;of\;novel\;object}{Total\;exploration\;time}\times 100$$

A more detailed description of the methodology is given in [45].

### 4.19. Thioflavin S Staining Procedure

Mice were anesthetized with isoflurane and transcardially perfused with 30 mL of cold PBS (pH = 7.4), followed by perfusion with 4% paraformaldehyde (BioVitrum, Saint Petersburg, Russia) in PBS. After that, the brain was extracted and immersed in 4% paraformaldehyde solution for a day. Subsequently, the brain samples were transferred to a 30% sucrose solution in PBS containing 0.02% sodium azide. The cerebral hemispheres were frozen in Neg 50 embedding medium, and frontal sections were made 20 µm thick on a Tissue-Tek Cryo3 microtome (Sakura Finetek, Torrance, CA, USA). Aβ plaques were stained for 5 min with a 1% solution of Thioflavin S diluted in 50% ethanol and counted using a LeicaDM 6000 CFS confocal scanning microscope (Leica Microsystems, Wetzlar, Germany). Data analysis was carried out in the entorhinal cortex and hippocampus at 10× magnification. The results of assessing the number of Aβ plaques were averaged over 8 sections of the brain of each animal.

### 4.20. Statistics

All data processing was performed using Microsoft Excel 2016^®^ and OriginPro 8.5. The results are expressed as the mean ± standard deviation. Statistical analysis of the results of in vivo experiments (determination of the number of Aβ plaques and behavioral test) was carried out using the Mann-Whitney test. Significance was tested at the 0.05 level of probability (*p*).

## 5. Conclusions

In conclusion, this study focused on the synthesis and self-organization of a new homologous series of dicationic gemini surfactants with two carbamate fragments (n-12-n(Et), where n represents the alkyl chain length, i.e., 10, 12, 14). Low cmc values (1, 0.11, and 0.013 mM obtained by tensiometry for 10-12-10(Et), 12-12-12(Et), 14-12-14(Et), respectively), high solubilizing capacity toward Orange OT (0.021, 0.029, 0.047 mol_OOT_/mol_GS_ for 10-12-10(Et), 12-12-12(Et), 14-12-14(Et), respectively), and high antimicrobial activity, especially toward resistant strains (minimum inhibitory concentration against methicillin-resistant strains of *S. aureus*, MRSA-1 and MRSA-2, is equal to 0.5 μg/mL for 10-12-10(Et)), and optimal ability to integrate into lipid bilayer were testified. In addition, a comparison between two series of surfactants, i.e., n-12-n(Et) and n-6-n(Et), revealed the advantage of carbamate surfactants with a dodecane spacer in terms of all the parameters studied. In the second part, soy phosphatidylcholine (PC) and cholesterol (Chol)-based liposomes were successfully modified with n-12-n(Et), which led to an increase in the zeta potential of liposomes (approximately + 40 mV), and as a result, ensured their stability up to 5 months. The cationic liposomes were loaded with antioxidant α-tocopherol and acetylcholinesterase inhibitor donepezil hydrochloride with an encapsulation efficiency of ≥90% for the treatment of Alzheimer’s disease in mice. It was found that the release of donepezil hydrochloride from PC/Chol/14-12-14(Et) liposomes follows a Fickian diffusion mechanism, which was determined using the Korsmeyer–Peppas release kinetic model. Moreover, these modified liposomes effectively reached the brains of rats in vivo via intranasal administration. In the final stage, it was demonstrated that treatment with PC/Chol/14-12-14(Et) liposomes loaded with α-tocopherol and donepezil hydrochloride for 21 days resulted in memory restoration of mice with Alzheimer’s disease model compared with untreated animals. Additionally, rate of Aβ plaque formation was reduced in transgenic mice brains in the entorhinal cortex, DG, and CA1 areas of the hippocampus. In this work, yet another promising liposomal drug delivery system modified with cationic GS is demonstrated, which serves as a ground to consider such systems for deeper and more complex in vivo tests.

## Figures and Tables

**Table 1:** The cmc values determined using tensiometry (tens) and conductometry (cond), as well as the maximum surface excess (Г_max_), surface area per surfactant molecule (A_min_), free energy of micellization (∆G_mic_), and the degree of counterion binding obtained by conductometry (β_cond_) and potentiometry (β_pot_) for n-12-n(Et), 298 K.

GS	cmc_tens_, mM	cmc_cond_, mM	Г_max_∙10^6^, mol∙m^−2^	A_min_, nm^2^	∆G_mic_, kJ∙mol^−1^	β_cond_	β_pot_
10-12-10(Et)	1 ± 0.03	1.5 ± 0.04	0.71	2.34	−25.5	0.41	-
12-12-12(Et)	0.11 ± 0.022	0.33 ± 0.01	0.41	4.08	−29.1	0.41	0.45
14-12-14(Et)	0.013 ± 0.0007	0.05 ± 0.0013	3.21	0.52	−37.0	0.44	0.3

**Table 2:** The cmc, solubilizing capacity (S) (toward Orange OT (OOT) and indomethacin (IND)), and aggregation number (N_agg_) values determined by fluorimetry and spectrophotometry at 298 K.

GS	cmc, mM		S, mol_probe_/mol_GS_		N_agg_ *
	Fluorimetry	Spectrophotometry	OOT	IND	
10-12-10(Et)	0.9 ± 0.03	1.5 ± 0.1	0.021	0.13	50
12-12-12(Et)	0.11 ± 0.002	0.16 ± 0.01	0.029	0.64	28
14-12-14(Et)	0.014 ± 0.0004	0.045 ± 0.003	0.047	1.51	16

* The N_agg_ of GS calculated using the Schott approach (for OOT solubilization).

**Table 3:** In vitro antibacterial and antifungal activities of GS. MIC—minimum inhibitory concentration, MBC—minimum bactericidal concentration, MFC—minimum fungicidal concentration.

GS	MIC, μg/mL									
	Gram-Positive Bacteria					Gram-Negative Bacteria		Fungi		
	*Sa*	*Bc*	*Ef*	*MRSA-1*	*MRSA-2*	*Ec*	*Pa*	*Ca*	*Tm*	*An*
10-12-10(Et)	0.5 ± 0.03	0.5 ± 0.04	0.5 ± 0.03	0.5 ± 0.03	0.5 ± 0.03	0.5 ± 0.03	1.9 ± 0.1	0.9 ± 0.07	0.9 ± 0.07	250 ± 19
12-12-12(Et)	0.9 ± 0.07	7.8 ± 0.6	1.9 ± 0.1	1.9 ± 0.1	0.9 ± 0.07	15.6 ± 1.3	125 ± 9	3.9 ± 0.3	3.9 ± 0.3	>250
14-12-14(Et)	3.9 ± 0.2	125 ± 10	3.9 ± 0.3	7.8 ± 0.6	7.8 ± 0.7	31.3 ± 2.5	125 ± 10	250 ± 19	250 ± 19	>250
Ketoconazole								3.9 ± 0.3	3.9 ± 0.3	15.6 ± 1.3
**MBC, μg/mL**								**MFC, μg/mL**		
10-12-10(Et)	0.5 ± 0.04	0.5 ± 0.03	0.5 ± 0.04	1.9 ± 0.1	3.9 ± 0.3	0.5 ± 0.03	3.9 ± 0.3	15.6 ± 1.2	15.6 ± 1.3	>250
12-12-12(Et)	0.9 ± 0.07	15.6 ± 1.3	3.9 ± 0.2	1.9 ± 0.1	0.9 ± 0.06	15.6 ± 1.2	125 ± 10	7.8 ± 0.6	7.8 ± 0.6	>250
14-12-14(Et)	3.9 ± 0.3	125 ± 9	3.9 ± 0.2	7.8 ± 0.6	7.8 ± 0.7	31.3 ± 2.6	125 ± 10	250 ± 21	250 ± 18	>250
Ketoconazole								3.9 ± 0.3	3.9 ± 0.3	125 ± 10

Sa—*S. aureus*, Bc—*B. cereus*, Ef—*E. faecalis*, MRSA-1 and MRSA-2—methicillin-resistant strains of *S. aureus*, Ec—*E. coli*, Pa—*P. aeruginosa*, Ca—*C. albicans*, Tm—*T. mentagrophytes*, An—*A. niger*.

**Table 4:** Physicochemical parameters of liposomes modified with 14-12-14(Et) at different concentrations of PC/Chol and surfactant/lipid ratio, 277 K. D_h_—hydrodynamic diameter, PdI—polydispersity index, ζ—zeta potential.

Lipid Concentration, mM	14-12-14(Et)/ Lipid Ratio	D_h_, nm	PdI	ζ, mV	D_h_, nm	PdI	ζ, mV
		1st Day			5th Month		
5	-	128 ± 1	0.063 ± 0.024	−1.6 ± 0.3	135 ± 17	0.414 ± 0.021	−23 ± 2
	1/100	120 ± 1	0.057 ± 0.029	13.8 ± 0.3	113 ± 4	0.346 ± 0.044	−24 ± 2
	1/50	116 ± 1	0.070 ± 0.003	27 ± 1	129 ± 1	0.135 ± 0.013	15 ± 1
	1/35	114 ± 1	0.070 ± 0.012	36 ± 3	126 ± 2	0.098 ± 0.024	27 ± 2
	1/25	113 ± 1	0.060 ± 0.003	46 ± 2	127 ± 1	0.175 ± 0.029	22.3 ± 0.5
10	-	129 ± 1	0.061 ± 0.024	0.8 ± 0.4	92 ± 1	0.216 ± 0.021	−22 ± 2
	1/100	112 ± 1	0.055 ± 0.019	13.7 ± 0.3	128 ± 1	0.127 ± 0.033	8 ± 1
	1/50	112 ± 1	0.070 ± 0.018	26.7 ± 0.8	119 ± 1	0.114 ± 0.017	25 ± 1
	1/35	112 ± 1	0.061 ± 0.013	34 ± 1	119 ± 1	0.095 ± 0.010	26 ± 1
	1/25	108 ± 1	0.069 ± 0.019	46 ± 2	114 ± 2	0.121 ± 0.034	22 ± 2
15	-	131 ± 2	0.069 ± 0.027	1.3 ± 0.4	103 ± 1	0.236 ± 0.010	−36 ± 6
	1/100	113 ± 1	0.055 ± 0.015	14.5 ± 0.7	134 ± 1	0.101 ± 0.005	14.7 ± 0.2
	1/50	113 ± 1	0.050 ± 0.023	26.4 ± 0.3	127 ± 2	0.112 ± 0.004	21 ± 3
	1/35	113 ± 1	0.062 ± 0.014	33 ± 1	125 ± 2	0.102 ± 0.024	32 ± 3
	1/25	108 ± 1	0.045 ± 0.012	43 ± 3	122 ± 2	0.139 ± 0.004	30 ± 1

**Table 5:** Physicochemical parameters of PC/Chol/n-12-n(Et) liposomes at different GS/lipid ratios, 277 K. Total PC/Chol concentration is 15 mM. D_h_—hydrodynamic diameter, PdI—polydispersity index, ζ—zeta potential.

GS	GS/Lipid Ratio	D_h_, nm	PdI	ζ, mV	D_h_, nm	PdI	ζ, mV
		1st Day			5th Month		
(PC/Chol)	-	131 ± 2	0.069 ± 0.027	1.3 ± 0.4	103 ± 1	0.236 ± 0.010	−36 ± 6
10-12-10(Et)	1/100	121 ± 1	0.049 ± 0.020	15 ± 1	131 ± 1	0.157 ± 0.017	8.1 ± 0.3
	1/50	117 ± 1	0.060 ± 0.010	28 ± 1	121 ± 1	0.105 ± 0.013	24 ± 1
	1/35	114 ± 1	0.061 ± 0.018	37 ± 1	118 ± 2	0.079 ± 0.014	28 ± 2
	1/25	112 ± 1	0.066 ± 0.010	45 ± 2	120 ± 1	0.097 ± 0.023	42 ± 2
12-12-12(Et)	1/100	119 ± 1	0.053 ± 0.035	17.5 ± 0.3	199 ± 4	0.393 ± 0.012	20.6 ± 0.6
	1/50	118 ± 1	0.072 ± 0.016	30 ± 2	185 ± 3	0.402 ± 0.050	33 ± 1
	1/35	119 ± 1	0.079 ± 0.016	40.6 ± 0.6	157 ± 1	0.328 ± 0.017	46 ± 1
	1/25	117 ± 1	0.081 ± 0.024	49 ± 2	149 ± 2	0.304 ± 0.041	49.8 ± 0.1
14-12-14(Et)	1/100	113 ± 1	0.055 ± 0.015	14.5 ± 0.7	134 ± 1	0.101 ± 0.005	14.7 ± 0.2
	1/50	113 ± 1	0.050 ± 0.023	26.4 ± 0.4	127 ± 2	0.112 ± 0.004	21 ± 3
	1/35	113 ± 1	0.062 ± 0.014	33 ± 1	125 ± 2	0.102 ± 0.024	32 ± 3
	1/25	108 ± 1	0.045 ± 0.012	43 ± 3	122 ± 2	0.139 ± 0.004	30 ± 1

**Table 6:** Physicochemical parameters of PC/Chol/n-12-n(Et) liposomes loaded with RhB, TOC, and DNP. GS/lipid ratio is 1/35, 277 K. EE—encapsulation efficiency, D_h_—hydrodynamic diameter, PdI—polydispersity index, ζ—zeta potential.

GS	PC/Chol Concentration, mM	EE, %	D_h_, nm	PdI	ζ, mV
RhB					
10-12-10(Et)	15	72 ± 1	103 ± 1	0.068 ± 0.018	33 ± 3
12-12-12(Et)	15	73.4 ± 0.8	105 ± 1	0.066 ± 0.009	36 ± 2
14-12-14(Et)	5	46 ± 2	110 ± 1	0.070 ± 0.014	36 ± 3
	10	66 ± 2	110 ± 1	0.079 ± 0.002	37 ± 2
	15	75 ± 3	110 ± 1	0.058 ± 0.015	35 ± 3
TOC					
10-12-10(Et)	15	95.8 ± 0.6	142 ± 2	0.124 ± 0.009	41 ± 2
12-12-12(Et)	15	91 ± 1	141 ± 2	0.077 ± 0.027	38 ± 2
14-12-14(Et)	5	94 ± 1	122 ± 1	0.074 ± 0.019	36 ± 2
	10	95 ± 1	133 ± 1	0.072 ± 0.002	38 ± 2
	15	96.1 ± 0.5	132 ± 1	0.081 ± 0.006	37 ± 2
DNP					
10-12-10(Et)	15	97.8 ± 0.6	107 ± 1	0.089 ± 0.024	41 ± 3
12-12-12(Et)	15	97.9 ± 0.4	109 ± 1	0.090 ± 0.013	50 ± 2
14-12-14(Et)	5	97.3 ± 0.4	109 ± 2	0.079 ± 0.018	32 ± 2
	10	97.6 ± 0.6	110 ± 2	0.105 ± 0.011	42 ± 1
	15	98.0 ± 0.5	110 ± 1	0.088 ± 0.005	40 ± 2

**Table 7:** hAChE and hBChE inhibitory concentrations and Selectivity index (SI) of GS in vitro.

GS	IC_50_ (hAChE), nM	IC_50_ (hBChE), nM	SI_hBChE/hAChE_
10-12-10(Et)	43.1 ± 0.9	19 ± 5	0.44
12-12-12(Et)	82 ± 13	61 ± 2	0.74
14-12-14(Et)	11.0 ± 0.5	1440 ± 520	131

**Table 8:** Adsorption and micellization parameters and equations.

Parameter	Equation
The surface excess (Г_max_)	Г_max_ = 1/(2.3nRT) × lim_c→CMC_ − (dπ/dlgC)
The minimum area per molecule (A_min_)	A_min_ = 10^18^/(*N*_A_ × Г_max_)
Free energy of micellization (ΔG_mic_)	∆G_mic_ = RT (0.5 + β) lnX_cmc_ − 0.5RTln2

R—the gas constant, π—value is equal to the difference between the surface tensions of the solvent and the amphiphile solution, T—the absolute temperature (K), n = 3 for dimeric surfactants, *N*_A_—the Avogadro number, the coefficient 10^18^ is used to convert the units from m^2^ to nm^2^, β—the degree of counterion binding, X_cmc_—mole fraction of cmc equals [cmc]/55.4.

## Data Availability

The analyzed data are included in this manuscript. Raw data are available from the authors upon request.

## Supplementary Materials

The following supporting information can be downloaded at: https://www.mdpi.com/article/10.3390/ijms241512312/s1.
