# Supplementary material for: Synthesis, Properties, and Biomedical Application of Dicationic Gemini Surfactants with Dodecane Spacer and Carbamate Fragments
Source: Int J Mol Sci. 2023 Aug 1;24(15):12312. doi: 10.3390/ijms241512312 (PMC10419252; doi:10.3390/ijms241512312)
Supplement: Supplementary file 1 [file ijms-24-12312-s001.zip › ijms-2538995-supplementary.pdf]

# Synthesis, Properties, and Biomedical Application of Dicationic Gemini Surfactants with Dodecane Spacer and Carbamate Fragments

**Leysan Vasileva, Gulnara Gaynanova, Farida Valeeva, Elvira Romanova, Rais Pavlov, Denis Kuznetsov, Grigory Belyaev, Irina Zueva, Anna Lyubina, Alexandra Voloshina, Konstantin Petrov and Lucia Zakharova ***

Arbuzov Institute of Organic and Physical Chemistry, FRC Kazan Scientific Center, Russian Academy of Sciences, 8 Arbuzov Str., 420088 Kazan, Russia
* Correspondence: luciaz@mail.ru

## 1. Synthesis

### 1.1. N,N'-didecyl-N,N'-bis(2-(ethylcarbamoyloxy)ethyl)-N,N'-dimethyldodecane-1,12-diammonium bromide, 10-12-10(Et).

Yield 0.61 g (68%). White solid. M.p. 107-110°C. **$^1$H NMR spectrum** (400 MHz, $CDCl_3$), δ, ppm (J, Hz): 0.87 t ($N^+$-$(CH_2)_9$-$\underline{CH}_3$, 6H, $^3J_{HH}$ 6.8); 1.16 t (-NH-$CH_2$-$\underline{CH}_3$, 6H, $^3J_{HH}$ 7.2); 1.40-1.26 two m (-$(\underline{CH}_2)_7$-$CH_3$, $N^+$-$(CH_2)_2$-$(\underline{CH}_2)_8$-$(CH_2)_2$-$N^+$, 44H); 1.73 m ($N^+$-$CH_2$-$\underline{CH}_2$-Alk, $N^+$-$CH_2$-$\underline{CH}_2$-$(CH_2)_8$-$\underline{CH}_2$-$CH_2$-$N^+$, 8H); 3.21 q (CO-NH-$\underline{CH}_2$-$CH_3$, 4H, $^3J_{HH}$ 7.1); 3.36 br. s ($N^+$-$\underline{CH}_3$, 6H); 3.45 m ($N^+$-$\underline{CH}_2$-$CH_2$-Alk, 4H); 3.57 m ($N^+$-$\underline{CH}_2$-$(CH_2)_{10}$-$\underline{CH}_2$-$N^+$, 4H); 3.87 m ($N^+$-$\underline{CH}_2$-$CH_2$-O-CO-, 4H); 4.54 m ($N^+$-$CH_2$-$\underline{CH}_2$-O-CO-, 4H). **IR** (KBr) ν: 3407, 3227, 2926, 2855, 1721, 0535, 1466, 1378, 1357, 1327, 1250, 1142, 1087, 1043, 992, 602, 844, 776, 722, 652, 585 cm$^{-1}$. **Elemental analysis** calcd for $C_{44}H_{92}Br_2N_4O_4$ (%): C 58.65; H 10.29; Br 17.74; N 6.22. Found (%): C 58.81; H 10.22; Br 17.65; N 6.30. **ESI-MS**: m/z = $[(M-2Br)/2]^{2+}$ 370.47 (calcd 370.28).

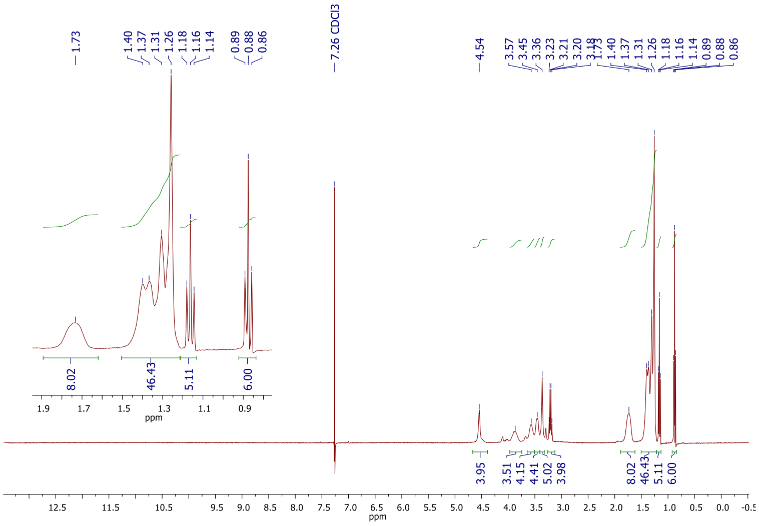


**Figure S1.** $^1$H NMR spectrum of 10-12-10(Et).

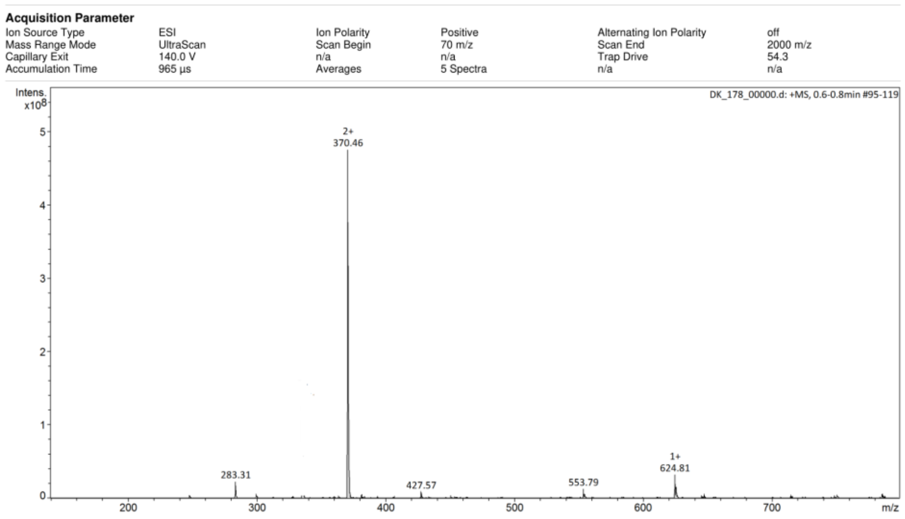


**Figure S2**. ESI mass spectrum of 10-12-10(Et).

### 1.2. N,N'-didodecyl-N,N'-bis(2-(ethylcarbamoyloxy)ethyl)-N,N'-dimethyldodecane-1,12-diammonium bromide, 12-12-12(Et).

Yield 0.55 g (57%). White solid. M.p. 124-127°C. **$^1$H NMR** spectrum (600 MHz, $CDCl_3$), δ, ppm (J, Hz): 0.88 t ($N^+$-$(CH_2)_{11}$-<u>CH</u>$_3$, 6H, $^3J_{HH}$ 6.9); 1.16 t (-NH-$CH_2$-<u>CH</u>$_3$, 6H, $^3J_{HH}$ 7.1); 1.40-1.25 two m (-(<u>CH</u>$_2$)$_9$-$CH_3$, $N^+$-$(CH_2)_2$-(<u>CH</u>$_2$)$_8$-$(CH_2)_2$-$N^+$, 52H); 1.76-1.72 m ($N^+$-$CH_2$-<u>CH</u>$_2$-Alk, $N^+$-$CH_2$-<u>CH</u>$_2$-$(CH_2)_8$-<u>CH</u>$_2$-$CH_2$-$N^+$, 8H); 3.21 q (CO-NH-<u>CH</u>$_2$-$CH_3$, 4H, $^3J_{HH}$ 7.1); 3.37 br. s ($N^+$-<u>CH</u>$_3$, 6H); 3.46 m ($N^+$-<u>CH</u>$_2$-$CH_2$-Alk, 4H); 3.58 m ($N^+$-<u>CH</u>$_2$-$(CH_2)_{10}$-<u>CH</u>$_2$-$N^+$, 4H); 3.90-3.85 m ($N^+$-<u>CH</u>$_2$-$CH_2$-O-CO-, 4H); 4.54 m ($N^+$-$CH_2$-<u>CH</u>$_2$-O-CO-, 4H). **IR** (KBr) ν: 3218, 3010, 2925, 2855, 1726, 1625, 1535, 1467, 1378, 1357, 1249, 1141, 1088, 1041, 988, 940, 904, 845, 777, 722, 661 $cm^{-1}$. **Elemental analysis** calcd for $C_{48}H_{100}Br_2N_4O_4$ (%): C 60.23; H 10.53; Br 16.70; N 5.85. Found (%): C 60.35; H 10.58; Br 16.84; N 5.79. **ESI-MS**: m/z = $[(M-2Br)/2]^{2+}$ 398.56 (calcd 398.31).

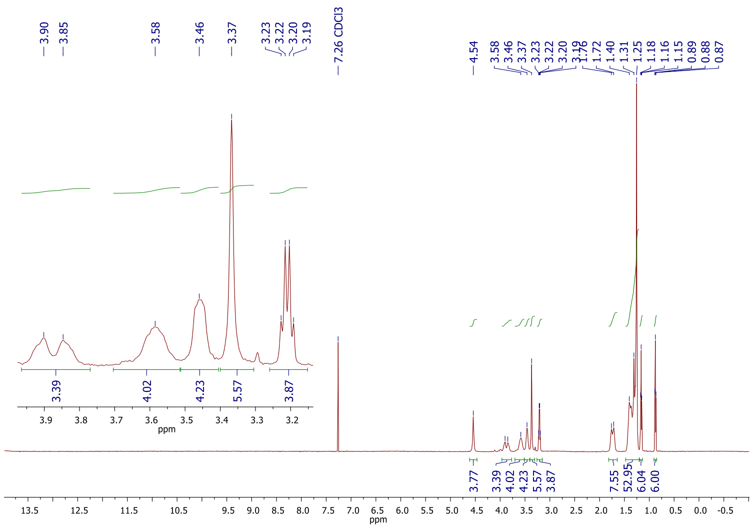


**Figure S3.** $^1$H NMR spectrum of 12-12-12(Et).

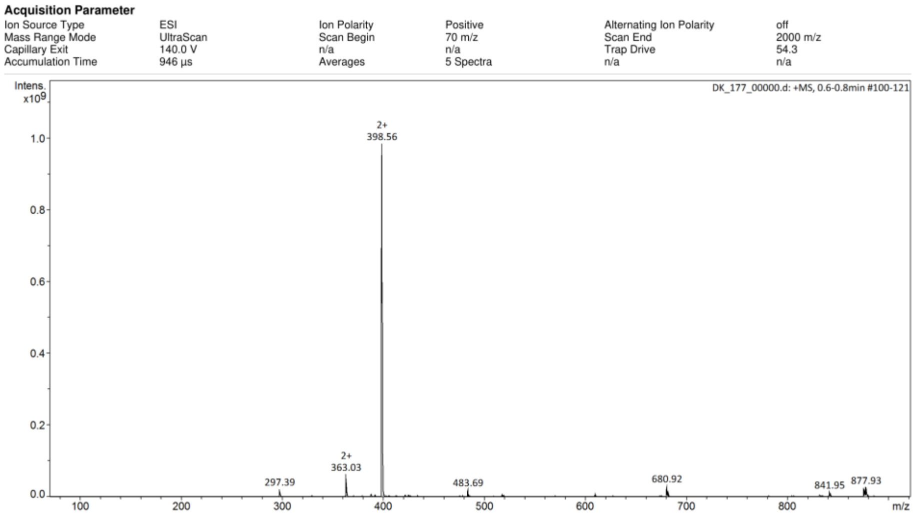


**Figure S4**. ESI mass spectrum of 12-12-12(Et).

**1.3. N,N'-bis(2-(ethylcarbamoyloxy)ethyl)-N,N'-dimethyl-N,N'-ditetradecyldodecane-1,12-diammonium bromide, 14-12-14(Et).**

Yield 0.54 g (53%). White solid. M.p. 120-123°C. **$^1$H NMR spectrum** (500 MHz, $CDCl_3$), δ, ppm (J, Hz): 0.85 t ($N^+$-$(CH_2)_{13}$-<u>CH</u>$_3$, 6H, $^3J_{HH}$ 6.8); 1.14 t (-NH-$CH_2$-<u>CH</u>$_3$, 6H, $^3J_{HH}$ 7.2); 1.36-1.23 two m (-(<u>CH</u>$_2$)$_{11}$-$CH_3$, $N^+$-$(CH_2)_2$-(<u>CH</u>$_2$)$_8$-$(CH_2)_2$-$N^+$, 60H); 1.71 m ($N^+$-$CH_2$-<u>CH</u>$_2$-Alk, $N^+$-$CH_2$-<u>CH</u>$_2$-$(CH_2)_8$-<u>CH</u>$_2$-$CH_2$-$N^+$, 8H); 3.18 q (CO-NH-<u>CH</u>$_2$-$CH_3$, 4H, $^3J_{HH}$ 7.2); 3.35 br. s ($N^+$-<u>CH</u>$_3$, 6H); 3.46 m ($N^+$-<u>CH</u>$_2$-$CH_2$-Alk, 4H); 3.56-3.54 m ($N^+$-<u>CH</u>$_2$-$(CH_2)_{10}$-<u>CH</u>$_2$-$N^+$, 4H); 3.89 m ($N^+$-<u>CH</u>$_2$-$CH_2$-O-CO-, 4H); 4.52 m ($N^+$-$CH_2$-<u>CH</u>$_2$-O-CO-, 4H). **IR** (KBr) ν: 3414, 3217, 3010, 2926, 2854, 1725, 1625, 1534, 1467, 1378, 1357, 1328, 1249, 1141, 1087, 1041, 992, 940, 903, 845, 776, 722, 663 $cm^{-1}$. **Elemental analysis** calcd for $C_{52}H_{108}Br_2N_4O_4$ (%): C 61.64; H 10.74; Br 15.77; N 5.53. Found (%): C 61.80; H 10.62; Br 15.71; N 5.65. **ESI-MS:** m/z = $[(M\text{-}2Br)/2]^{2+}$ 426.57 (calcd 426.34).

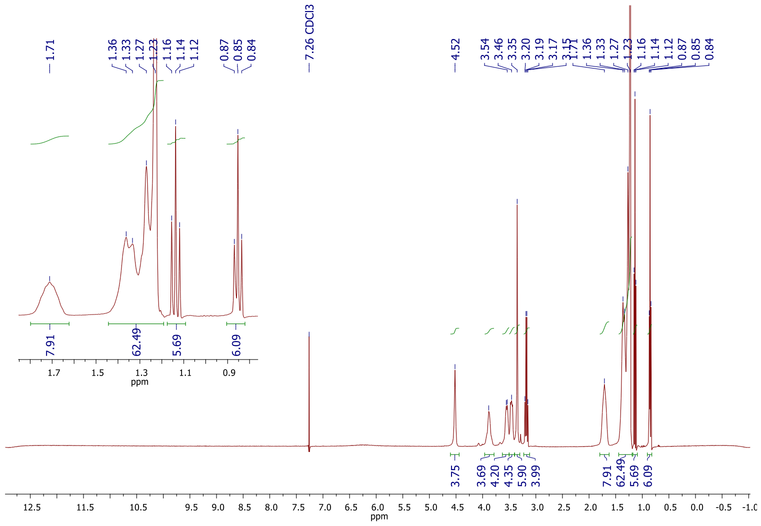


**Figure S5.** $^1$H NMR spectrum of 14-12-14(Et).

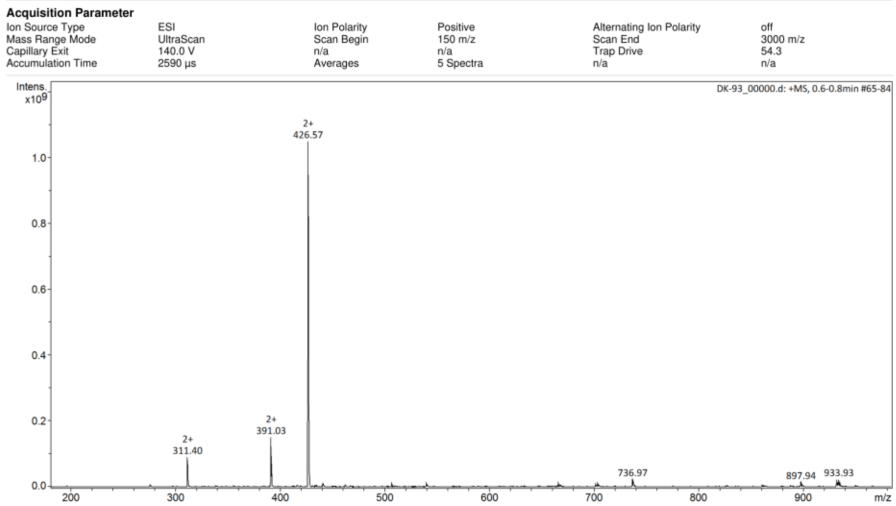


**Figure S6**. ESI mass spectrum of 14-12-14(Et).

## 2. Potentiometry

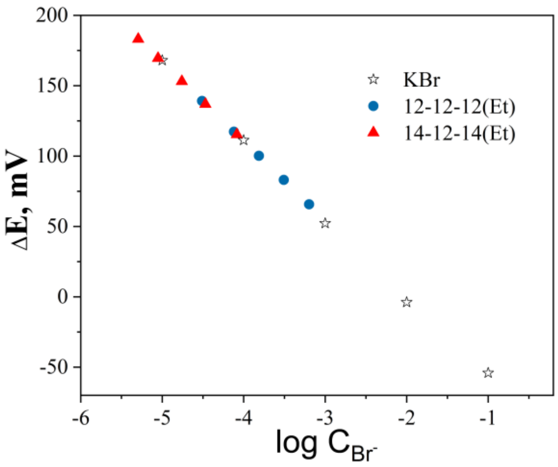


**Figure S7.** Change in electrode potential ($\Delta E$) on bromide counterion concentration for KBr, 12-12-12(Et), and 14-12-14(Et) aqueous solutions, 298 K. The Nernst slope for KBr solutions is 56 mV equiv$^{-1}$.

## 3. Fluorescence spectroscopy data

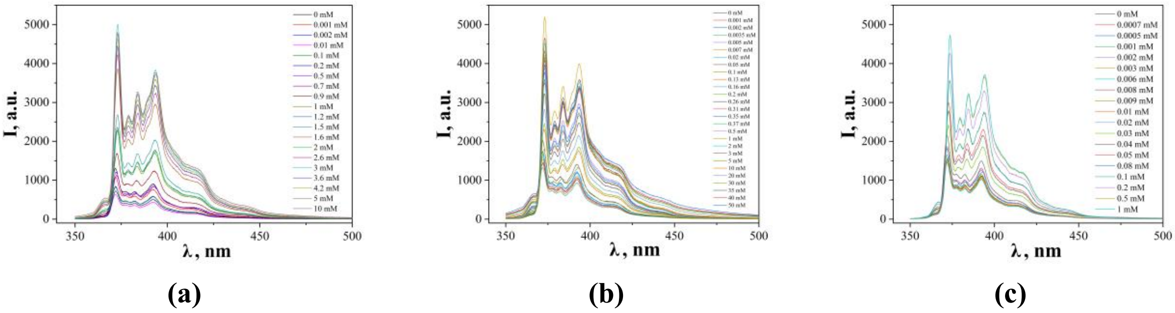


**Figure S8.** Fluorescence spectra of 1 µM pyrene in (a) 10-12-10(Et); (b) 12-12-12(Et) and (c) 14-12-14(Et) surfactant solutions, 298 K.

**Table S1.** Values of DPH anisotropy (r) depending on the GS concentration, 298 K.

| GS | $C_{GS}$, mM | r |
|---|---|---|
| 10-12-10(Et) | 1.2 | 0.133 |
| | 2.2 | 0.126 |
| | 2.6 | 0.090 |
| 12-12-12(Et) | 0.31 | 0.134 |
| | 0.37 | 0.108 |
| | 0.5 | 0.089 |
| | 0.8 | 0.072 |
| 14-12-14(Et) | 0.06 | 0.127 |
| | 0.1 | 0.114 |
| | 0.5 | 0.092 |
| | 1 | 0.072 |

## 4. Spectrophotometry data

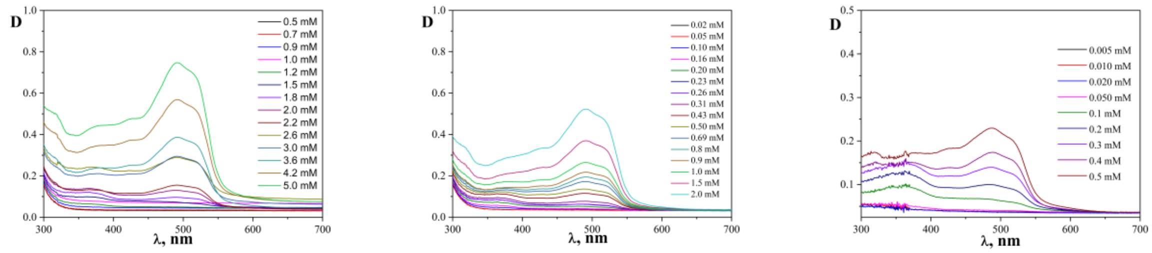


**(a)** **(b)** **(c)**

**Figure S9**. Absorption spectra of Orange OT in micellar solutions of (a) 10-12-10(Et); (b) 12-12-12(Et); (c) 14-12-14(Et), 298 K, l = 0.5 cm.

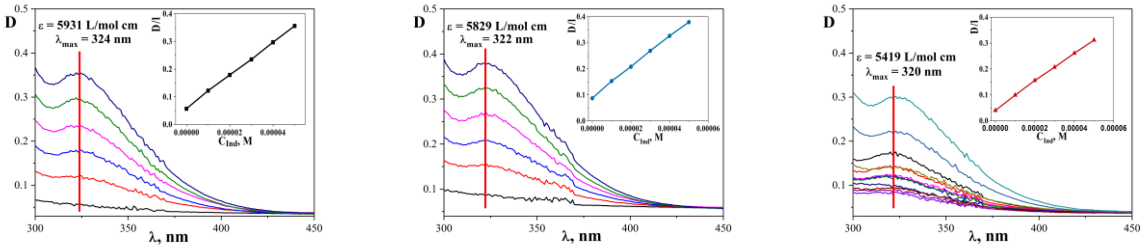


**(a)** **(b)** **(c)**

**Figure S10.** Absorption spectra of indomethacin in aqueous solutions of (a) 10-12-10(Et); (b) 12-12-12(Et); (c) 14-12-14(Et), 298 K, l = 1 cm.

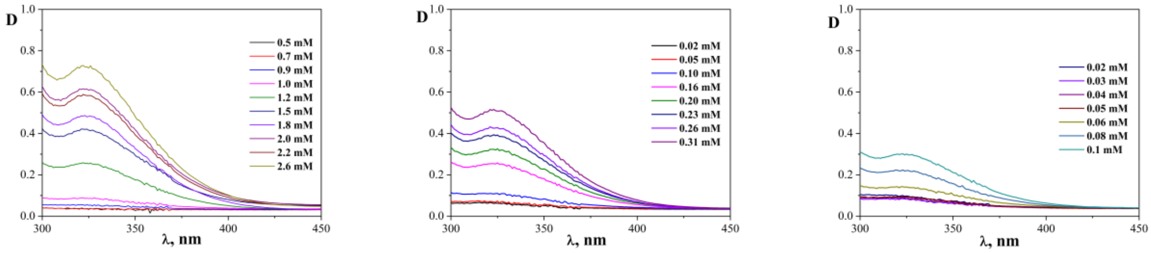


**(a)** **(b)** **(c)**

**Figure S11.** Absorption spectra of indomethacin in micellar solutions of (a) 10-12-10(Et); (b) 12-12-12(Et); (c) 14-12-14(Et), 298 K, l = 0.5 cm.

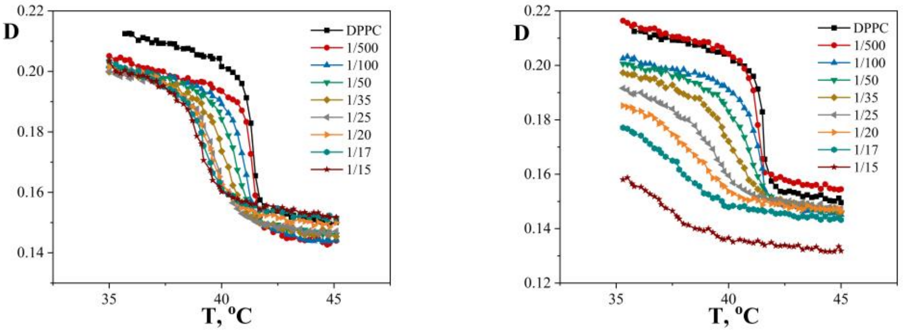


**(a)** **(b)**

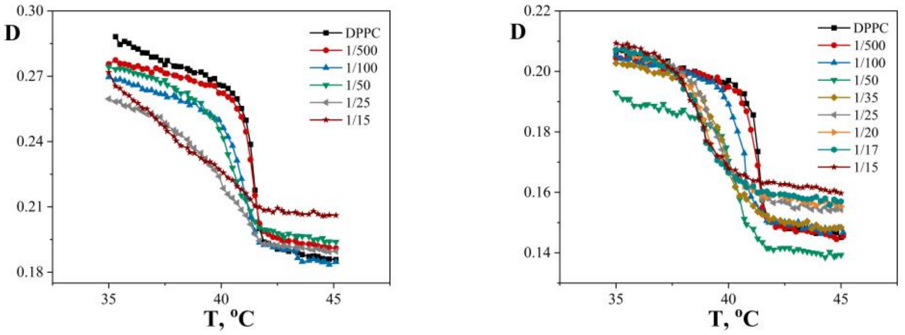


**(c)** **(d)**

**Figure S12.** Turbidimetric plots for (a) 10-12-10(Et)/DPPC; (b) 12-12-12(Et)/DPPC; (c) 14-12-14(Et)/DPPC; (d) 12-6-12(Et)/DPPC binary systems at various surfactant/lipid molar ratio.

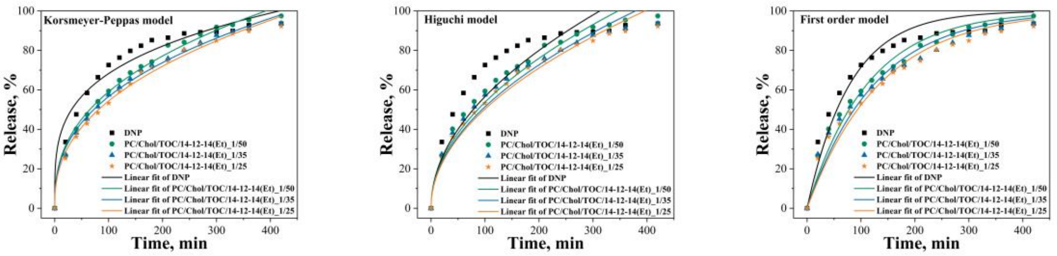


**(a)** **(b)** **(c)**

**Figure S13.** The release kinetic model fitting curves of free DNP and DNP encapsulated in PC/Chol/TOC/14-12-14(Et) (1/50, 1/35, 1/25): (a) Korsmeyer–Peppas model; (b) Higuchi model;

(c) first order kinetic model. Total lipid concentration is 15 mM. Phosphate buffer (0.025 M), pH = 7.4, 310 K.

**Table S2.** The release kinetic model fitting parameters of free DNP and DNP encapsulated in liposomes.

| System | Model | | | | | | |
|---|---|---|---|---|---|---|---|
| | **Korsmeyer-Peppas** | | | **Higuchi** | | **First order** | |
| | n | $k_{KP}$, %/min$^n$ | $R^2$ | $k_H$, %/min$^{1/2}$ | $R^2$ | $k_1$, 1/min | $R^2$ |
| DNP | 0.268 ± 0.023 | 19.88 ± 2.48 | 0.9636 | 5.66 ± 0.22 | 0.7540 | 0.0125 ± 0.0008 | 0.9372 |
| PC/Chol/14-12-14(Et) 1/35* | 0.379 ± 0.016 | 9.82 ± 0.84 | 0.9892 | 5.09 ± 0.10 | 0.9495 | 0.0078 ± 0.0004 | 0.9544 |
| PC/Chol/TOC/14-12-14(Et) 1/50* | 0.386 ± 0.013 | 9.96 ± 0.71 | 0.9926 | 5.37 ± 0.10 | 0.9583 | 0.0089 ± 0.0004 | 0.9696 |
| PC/Chol/TOC/14-12-14(Et) 1/35* | 0.383 ± 0.011 | 9.68 ± 0.56 | 0.9950 | 5.14 ± 0.09 | 0.9586 | 0.0079 ± 0.0004 | 0.9533 |
| PC/Chol/TOC/14-12-14(Et) 1/25* | 0.405 ± 0.013 | 8.41 ± 0.60 | 0.9931 | 5.02 ± 0.08 | 0.9707 | 0.0074 ± 0.0003 | 0.9617 |

$k_{KP}$ is the release constant taking into account the structural and geometric characteristics of the dosage form; n is the diffusion release exponent; $k_H$ is the Higuchi release constant; $k_1$ is the first order release constant.
*Total lipid concentration is 15 mM.

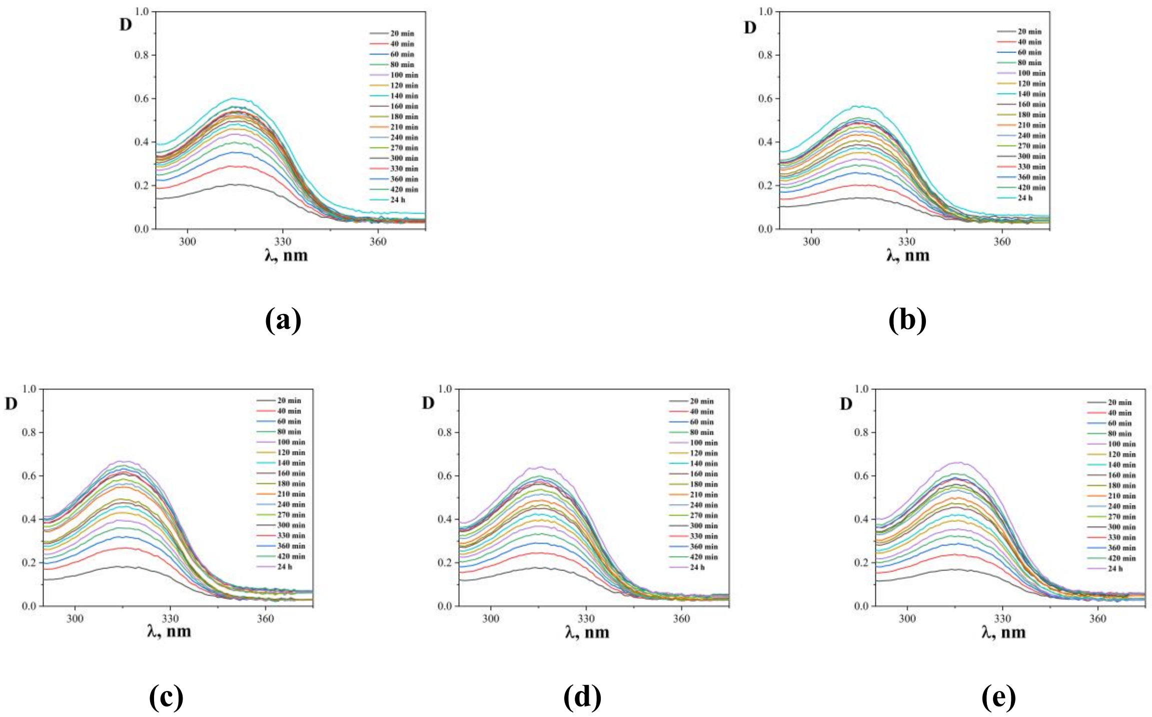


**Figure S14.** The absorption spectra of DNP at different time intervals of release: (a) free DNP; (b) PC/Chol/14-12-14(Et)/DNP 1/35; (c) PC/Chol/TOC/14-12-14(Et)/DNP 1/50; (d) PC/Chol/TOC/14-12-14(Et)/DNP 1/35; (e) PC/Chol/TOC/14-12-14(Et)/DNP 1/25. Total lipid concentration is 15 mM. Phosphate buffer (0.025 M), pH = 7.4, 310 K, cuvette thickness = 1 cm.
